# Novel enhancement mechanism of tyrosine hydroxylase enzymatic activity by nitric oxide through S-nitrosylation

**DOI:** 10.1038/srep44154

**Published:** 2017-03-13

**Authors:** Yuanyuan Wang, Chun Chau Sung, Kenny K. K. Chung

**Affiliations:** 1 Division of Life Science, State Key Laboratory of Molecular Neuroscience, The Hong Kong University of Science and Technology, Hong Kong, China

## Abstract

Tyrosine hydroxylase (TH) is a rate-limiting step enzyme in the synthesis of catecholamines. Catecholamines function both as hormone and neurotransmitters in the peripheral and central nervous systems, therefore TH’s expression and enzymatic activity is tightly regulated by various mechanisms. Several post-translational modifications have been shown to regulate TH’s enzymatic activity such as phosphorylation, nitration and S-glutathionylation. While phosphorylation at N-terminal of TH can activate its enzymatic activity, nitration and S-glutathionylation can inactivate TH. In this study, we found that TH can also be S-nitrosylated by nitric oxide (NO). S-nitrosylation is a reversible modification of cysteine (cys) residue in protein and is known to be an emerging signaling mechanism mediated by NO. We found that TH can be S-nitrosylated at cys 279 and TH S-nitrosylation enhances its enzymatic activity both *in vitro* and *in vivo*. These results provide a novel mechanism of how NO can modulate TH’s enzymatic activity through S-nitrosylation.

Tyrosine hydroxylase (TH) is a rate-limiting step enzyme in the production of catecholamines such as dopamine (DA), epinephrine and norepinephrine (NE). Being the rate-limiting enzyme in the first step of catecholamine synthesis, it converts tyrosine to L-dihydroxyphenyalanine (L-DOPA) in which L-DOPA will be further converted to DA by DOPA decarboxylase^1^,^2^. DA can then further convert to NE by dopamine β-hydroxylase (DBH) and NE can be converted to epinephrine by phentolamine N-methyltransferase (PNMT)^1^,^2^. Catecholamines such as NE and epinephrine function as hormone and neurotransmitter in the peripheral nervous system which regulate a lot of vital physiological functions. In the central nervous system, catecholamines also serve as an important category of neurotransmitter that control major cognitive functions such as emotion, learning and memory^1^,^2^. Because of the critical role of catecholamines in maintaining the normal physiological functions, the expression and enzymatic activity of TH are tightly regulated. Particularly for short time changes in neuronal activity, the TH activity will need to modulate fast to meet the local demand^3^,^4^,^5^,^6^. For instance, the TH enzymatic activity has been shown to be regulated by a number of post-translational modifications (PTM) such as phosphorylation, ubiquitination, nitration and S-glutathionylation^2^,^7^,^8^. Studies have found that TH phosphorylation can increase its enzymatic activity while nitration and S-glutathionylation cause loss of activity^2^,^3^,^7^,^8^. From this study, we report a novel PTM of TH through S-nitrosylation by nitric oxide (NO). S-nitrosylation is a reversible modification of cysteine (cys) residue in proteins that recently has shown to regulate increasing number of cellular pathways^9^,^10^. From our preliminary study in the identification of potential S-nitrosylated proteins in dopaminergic system, we found that TH can be S-nitrosylated both *in vitro* and *in vivo*. We further found that TH is S-nitrosylated at cys 279 and this enhances its enzymatic activity. In addition, we found that treatment of mice with DA receptor agonists, such as apomorphine, can enhance TH’s enzymatic activity through S-nitrosylation. These results show that TH enzymatic activity can be modulated by a novel mechanism through S-nitrosylation by NO.

## Results

### Tyrosine hydroxylase is S-nitrosylated both *in vitro* and *in vivo*

**Figure 1:**
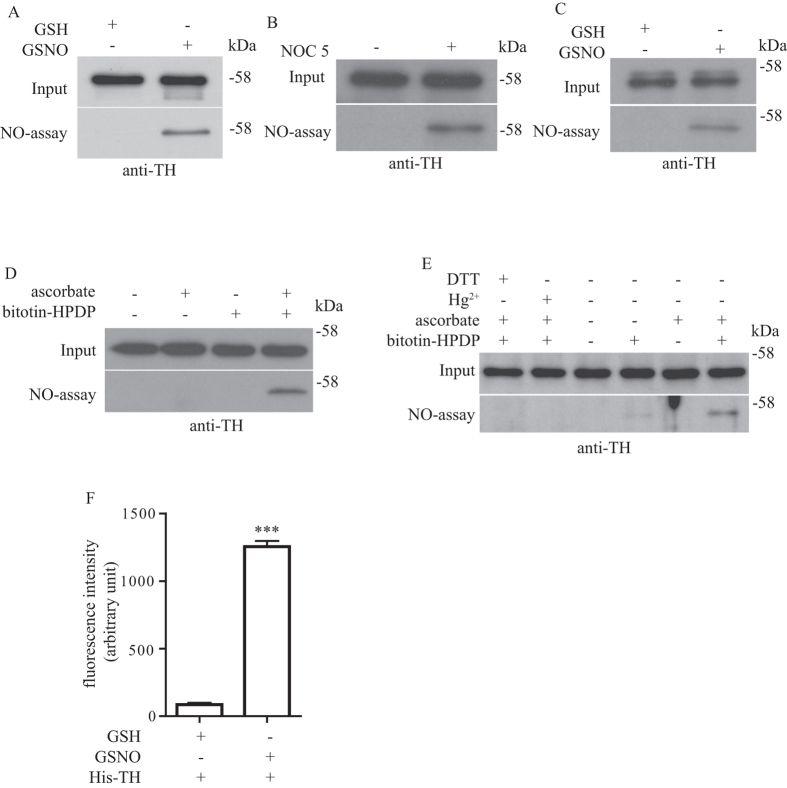
TH is S-nitrosylated both *in vitro* and *in vivo*. (**A**) Cell lysates from HEK293T cells expressing human TH were treated with 250 μM GSH or GSNO and then subjected to biotin-switch assay. TH was S-nitrosylated after GSNO treatment. (**B**) Cell lysates from HEK293T cells expressing human TH were treated with or without 250 μM NOC5 and then subjected to biotin-switch assay. TH was S-nitrosylated after NOC5 treatment. (**C**) Cell lysates from PC12 cells were treated with 250 μM GSH or GSNO and then subjected to biotin-switch assay. PC12 cells endogenous TH was S-nitrosylated after GSNO treatment. **(D)** Mouse brain striatum tissue lysates were subjected to *in vivo* biotin-switch assay. Samples were treated with or without or in combination of ascorbate and biotin-HPDP as indicated. Only in the presence of ascorbate and biotin-HPDP could S-nitrosylated TH be detected, showing that TH was S-nitrosylated *in vivo* in the striatum. (**E**) Similar experiment was performed as in (D) but with the additional sample pretreatment with 200 μM of DTT or Hg^2+^ (**F**) Recombinant his-tagged TH was treated with 250 μM of GSH or GSNO and subjected to fluorometric DAN assay. NO could be released from TH after GSNO treatment and could be detected by the fluorometric method (***p < 0.001; n = 3). Figure 1A–E included cropped images and the original blot images are included in the Supplementary Information file.

From our preliminary screening of S-nitrosylation of proteins in the dopaminergic system, we identified a potential candidate, TH which is a rate limiting step enzyme that could be post-translationally modified through S-nitrosylation by nitric oxide (NO). To perform a more systematic study on how TH S-nitrosylation could modulate its function, we first cloned TH to determine if it could be S-nitrosylated *in vitro*. We expressed recombinant TH in HEK293T cells and treated the cell lysates with NO donors GSNO or NOC5 and then performed the biotin-switch assay to determine if TH could be S-nitrosylated. We found that prominent TH S-nitrosylation could be detected after both GSNO and NOC5 treatment (Fig. 1A and B). To further confirm that TH could be S-nitrosylated in cells normally expressing TH, we used PC12 cell lysates and treated with GSNO. We then performed the biotin-switch assay and found that endogenous TH expressed in PC12 cells could also be S-nitrosylated by NO (Fig. 1C). To determine if TH could be S-nitrosylated *in vivo*, we harvested striatum from mice and perform *in vivo* biotin-assay and found that TH can be S-nitrosylated *in vivo* (Fig. 1D). To further confirm that TH can be S-nitrosylated *in vivo*, we prepared the brain samples with either DTT (Dithiothreitol) or Hg^2+^, two treatments that are well-known to release NO from S-nitrosylated proteins, and performed an assay as in Fig. 1D. We found that both treatment of DTT and Hg^2+^ could reverse TH S-nitrosylation in brain samples (Fig. 1E). To use an alternative method to confirm TH could be S-nitrosylated, we treated recombinant TH with GSNO and then released the NO moiety with Hg^2+^ and detected the NO released by the fluorometric DAN assay. In agreement with our biotin-switch assay, NO could be released from TH showing that this is a reversible modification (Fig. 1F).

### TH S-nitrosylation enhances its enzymatic activity by increasing its V_max_

**Figure 2:**
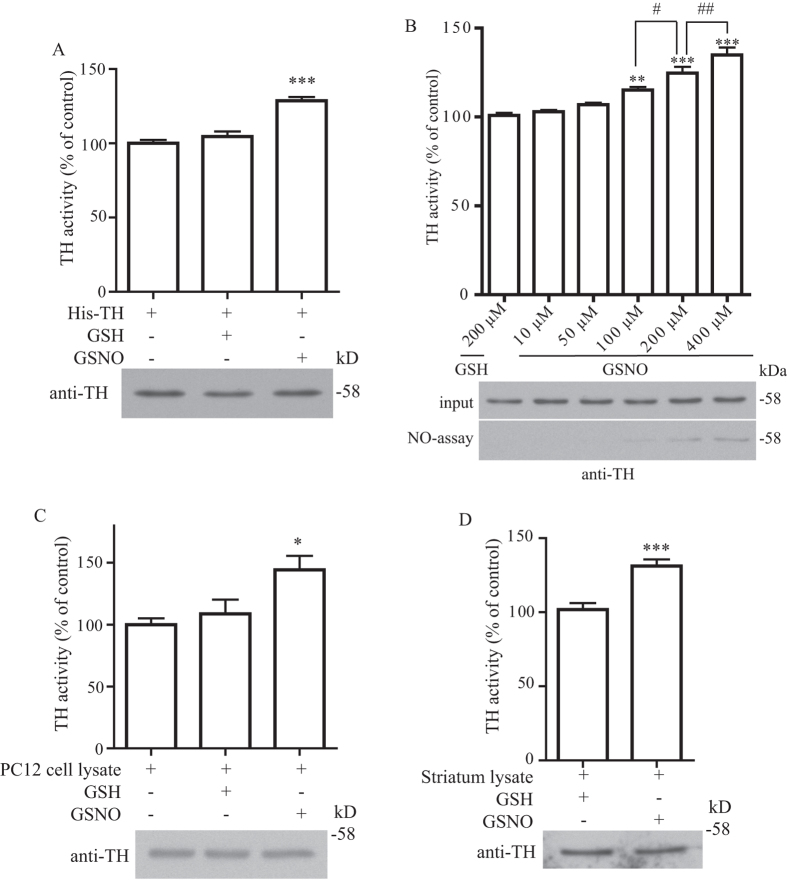
TH S-nitrosylation enhances its enzymatic activity. (**A**) Recombinant his-tagged TH was treated with 200 μM GSH or GSNO and then subjected to real-time enzymatic kinetic assay. Treatment of GSNO significantly enhanced TH’s enzymatic activity (***p < 0.001; n = 9). (**B**) Recombinant his-tagged TH was treated with 200 μM of GSH or different concentration of GSNO and then subjected to real-time enzymatic kinetic assay or biotin-switch assay. Both enhanced in TH enzymatic activity and amount of TH S-nitrosylation were dose-dependent on GSNO treatment (**p < 0.01 and ***p < 0.001 vs. the 200 μM GSH group; ^#^p < 0.05 and ^##^p < 0.01 vs. the 200 μM GSNO group as indicated; n = 3). (**C**) PC12 cell lysates were treated with 200 μM GSH or GSNO and then subjected to real-time enzymatic kinetic assay. Treatment of GSNO significantly enhanced the endogenous PC12 cell’s TH enzymatic activity (*p < 0.05; n = 9). **(D)** Brain tissue lysates from mouse striatum were treated with 200 μM GSH or GSNO and then subjected to real-time enzymatic kinetic assay. Treatment of GSNO significantly enhanced the TH enzymatic activity in the mouse brain striatum (***p < 0.001; n = 9). Figure 2A–D included cropped images and the original blot images are included in the Supplementary Information file.

Since we found that TH can be S-nitrosylated both *in vitro* and *in vivo*, we suspected that TH S-nitrosylation can modulate its enzymatic activity. To determine if TH S-nitrosylation can modulate its enzymatic activity, we employed a real-time enzymatic kinetic assay^11^ to monitor recombinant TH’s enzymatic activity with or without S-nitrosylation. We found that treatment of TH with GSNO significantly increased its enzymatic activity (Fig. 2A). To determine if GSNO induced TH increased in enzymatic activity was dose-dependent, we treated TH with different concentration of GSNO and performed both enzymatic kinetic assay and biotin-switch assay. We found that both amount of TH S-nitrosylation and its enzymatic activity changes were depended on the concentration of GSNO treatment (Fig. 2B). To further confirm that this enhanced enzymatic activity by NO was not specific to recombinant TH, we used PC12 lysates to perform similar experiment and found that GSNO treatment could also significantly increase the endogenous TH activity in PC12 lysates (Fig. 2C). To determine if TH activity from mouse brain striatum could also be modulated by NO, we treated mouse brain striatum lysates with GSNO and performed the TH enzymatic kinetic assay. In agreement with our recombinant and PC12 TH enzymatic results, treatment of mouse brain striatum lysates with GSNO also enhanced the enzymatic activity of TH (Fig. 2D).

**Figure 3:**
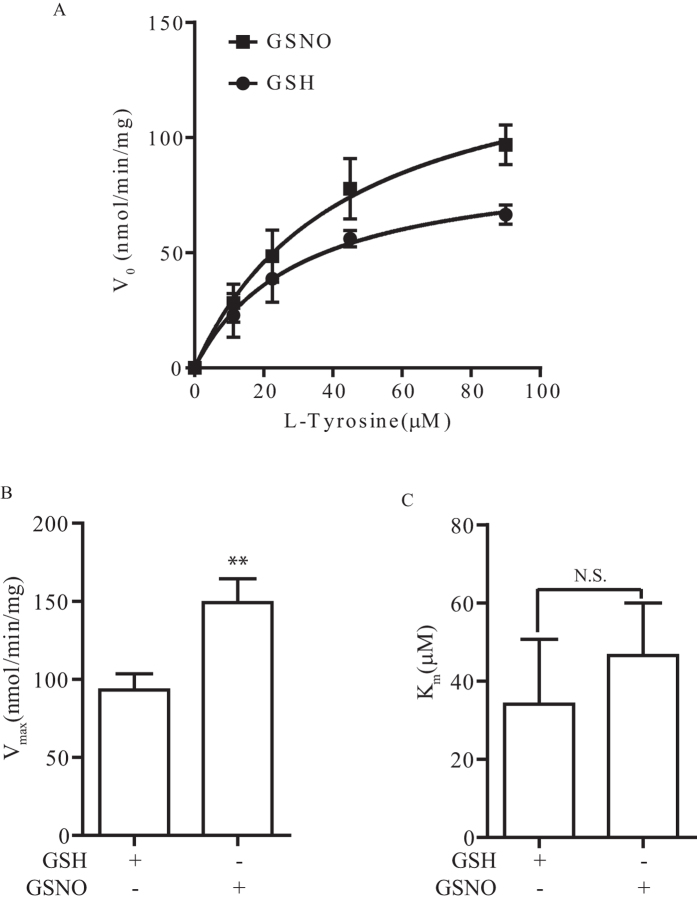
TH S-nitrosylation enhanced its enzymatic activity through increasing V_max_. (**A**) Recombinant his-tagged TH was first treated with 200 μM GSH or GSNO and reaction rate of TH incubated with different concentrations of L-tyrosine was determined (n = 3). (**B**) Calculation of V_max_ from (**A**) showed that treatment of GSNO significantly increased the V_max_ of TH (**p < 0.01; n = 3). (**B**) Calculation of K_m_ from (**A**) showed that treatment of GSNO did not affect the Km of TH (N.S.; no significant; n = 3).

To determine the potential mechanism of how TH S-nitrosylation can modulate its enzymatic activity, we measured the maximal velocity (V_max_) and the Michaelis Constant (K_m_) of TH with or without S-nitrosylation with a well-established method as described in the materials and method section^12^,^13^. We incubated TH treated with GSH or GSNO with different concentrations of L-tyrosine and measure the rate of reaction of TH converting L-tryrosine to L-DOPA (Fig. 3A). We then used the data and calculated the V_max_ and K_m_ of TH treated with GSH or GSNO. We found that TH S-nitrosylation significantly increased its V_max_ (Fig. 3B) but the K_m_ was not affected (Fig. 3C). This results suggested that TH S-nitrosylation enhanced its enzymatic activity by increasing the rate of reaction (V_max_) without affecting its affinity for substrate binding (K_m_).

### TH is S-nitrosylated at cys 279 and this PTM enhances its enzymatic activity

**Figure 4:**
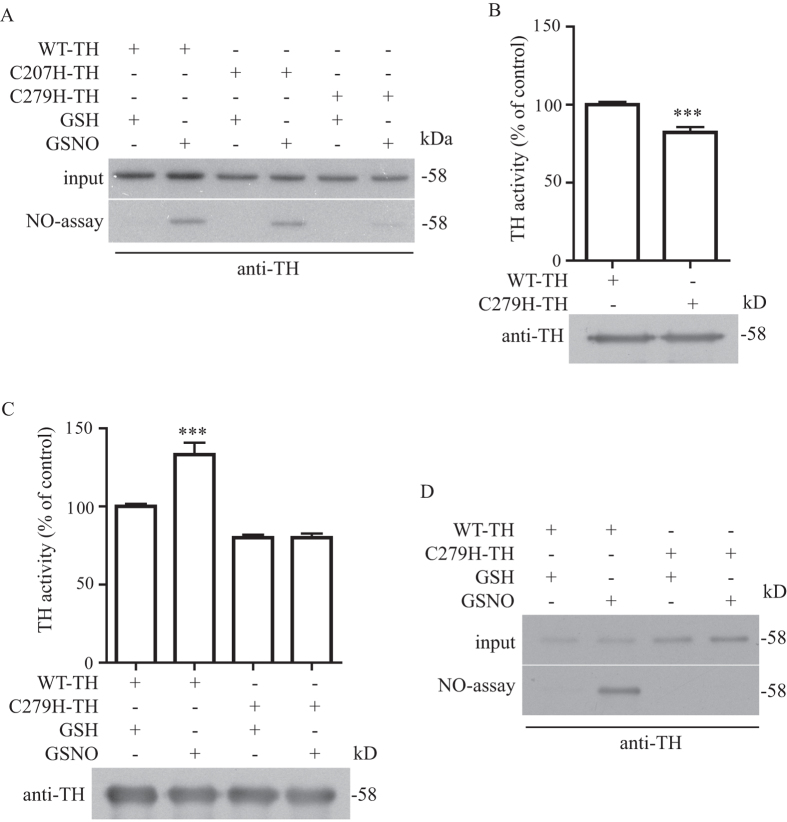
TH is S-nitrosylated at cys 279 and enhances its enzymatic activity. (**A**) Site mutagenesis was performed and cys 279 of TH was found to be site of TH S-nitrosylation. Biotin-switch assay was performed on WT, C207H and C279H TH expressed in HEK293T cells, C279H TH was found to have a reduction on TH S-nitrosylation. (**B**) WT and C279H TH were subjected to real-time enzymatic kinetic assay. C279H TH has a significant enzymatic activity as compared to WT (***p < 0.001; n = 6). (**C**) WT and C279H TH recombinant proteins were subjected to real-time enzymatic kinetic assay after treatment with 200 μM GSH or GSNO. Treatment GSNO in WT TH significantly enhanced the TH’s enzymatic activity but this modulation was abrogated in C279H TH (***p < 0.001; n = 9). (**D**) WT and C279H TH recombinant proteins were subject to biotin-switch assay. The mutation in cys 279 abrogated S-nitrosylation in C279H TH recombinant proteins. Figure 4A–D included cropped images and the original blot images are included in the Supplementary Information file.

To identify the site of TH S-nitrosylation, we performed site mutagenesis study and found that cys 279 is the site of TH S-nitrosylation as shown by the biotin-switch assay (Fig. 4A). By comparison, we found that C279H-TH had a reduction of S-nitrosylation as compared to WT-TH and C207H-TH (Fig. 4A). To determine if S-nitrosylation of TH at cys 279 is responsible for the enhancement of TH enzymatic activity by NO, we performed real-time enzymatic kinetic assay on the WT-TH and the C279H-TH recombinant proteins and found that mutation at cys 279 of TH significantly reduced its enzymatic activity (Fig. 4B), indicating that this is an important amino acid residue that can regulate the TH’s enzymatic activity. To further determine if S-nitrosylation of TH cys 279 can modulate TH’s enzymatic activity, we performed the real-time enzymatic kinetic assay on the WT-TH and The C279H-TH recombinant proteins in the presence or absence of NO donor GSNO (Fig. 4C). In agreement with our previous results, GSNO treatment could enhance the TH enzymatic activity of WT-TH, but this enhancement was completely abrogated in C279H-TH (Fig. 4C), suggesting that cys 279 is the site of TH S-nitrosylation which enhances its enzymatic activity. To determine if this cys 279 mutation in this recombinant TH protein would also affected its S-nitrosylation, we performed biotin-switch assay and found that mutation of cys 279 also abrogated S-nitrosylation in this mutant (Fig. 4D).

### TH enzymatic activity is enhanced by S-nitrosylation during the activation of dopaminergic transmission *in vivo*

**Figure 5:**
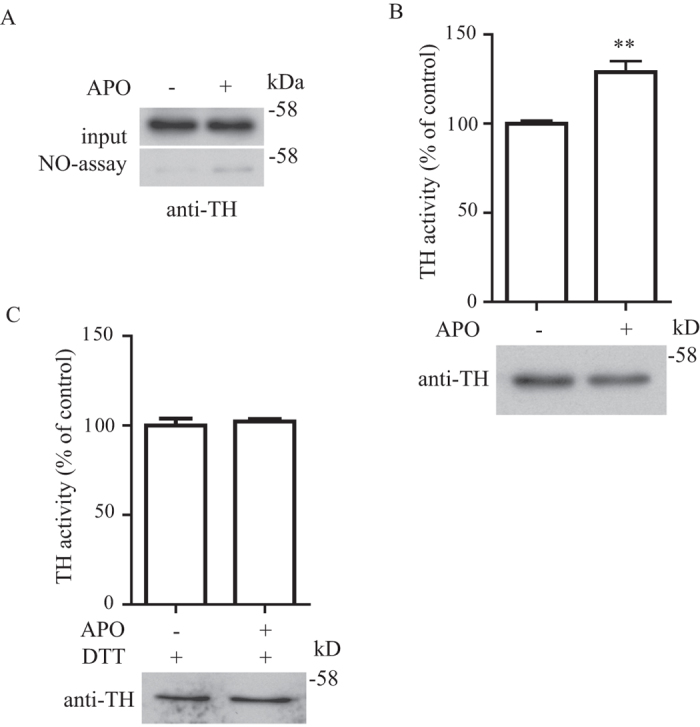
TH enzymatic activity is enhanced by S-nitrosylation during the activation of dopaminergic transmission *in vivo*. **(A)** Striatum from mice treated with or without apomorphine (APO) was subjected to biotin-switch assay. Treatment of APO increased the S-nitrosylation of TH in the mouse striatum. (**B**) Brain striatum tissue lysates isolated from mice treated with APO have significant enhanced TH enzymatic activity (**p < 0.01; n = 8). **(C)** Similar experiments as in (**B**) were performed except samples were pretreated with 200 μM of DTT to reverse the NO modification. Treatment of DTT abrogated the effect of APO treatment (n = 6). Figure 5A–C included cropped images and the original blot images are included in the Supplementary Information file.

**Figure 6:**
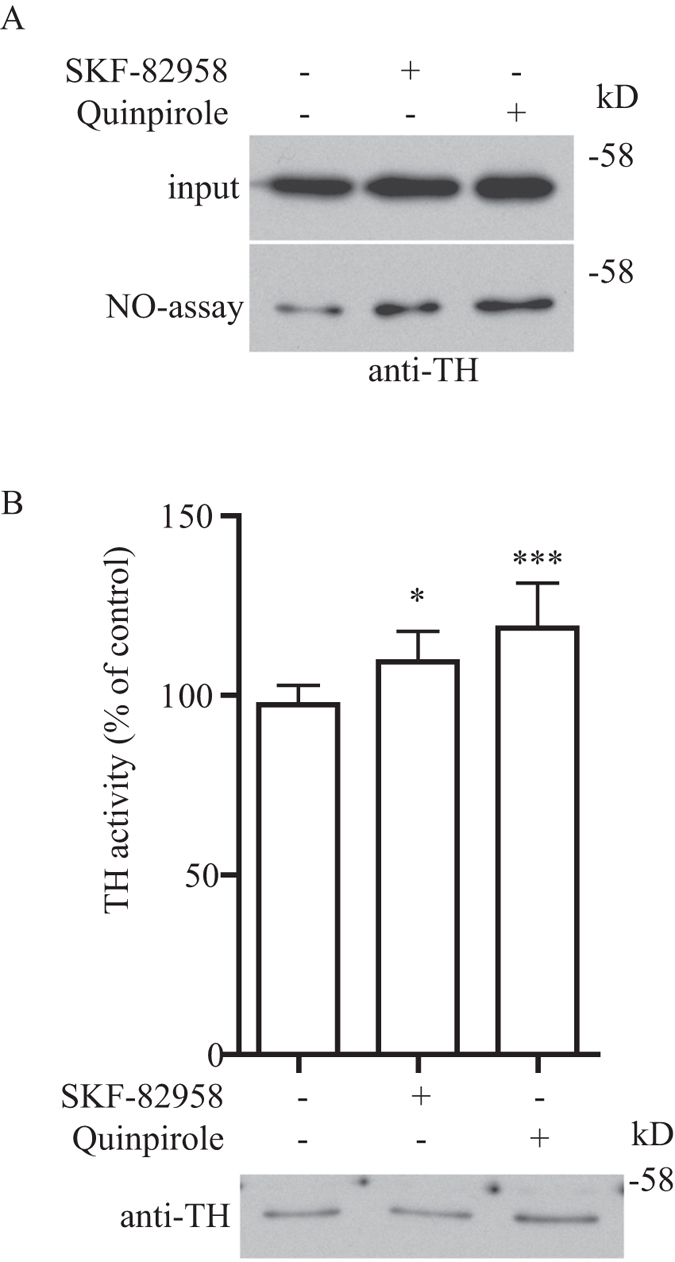
TH enzymatic activity is enhanced by S-nitrosylation through activation of both DA receptor D1 and D2. (**A**) Brain striatum tissue lysates isolated from mice treated with control, SKF-82958 (D1 agonist) or quinpirole (D2 agonist) was subjected to biotin-switch assay. Treatment of both SKF-82958 and quinpirole increased the S-nitrosylation of TH in the mouse striatum. **(B)** Brain striatum tissue lysates isolated from mice treated with control, SKF-82958 (D1 agonist) or quinpirole (D2 agonist) was subjected to TH real-time enzymatic kinetic assay. Treatment of both SKF-82958 and quinpirole increased TH enzymatic activity in the mouse striatum (*p < 0.05; **p < 0.01; n = 9). Figure 6A,B included cropped images and the original blot images are included in the Supplementary Information file.

To determine if TH enzymatic activity enhancement by S-nitrosylation is relevant in neuronal signal transduction *in vivo*, we employed an established mouse model to activate dopaminergic transmission by the injection of non-selective DA receptor agonist apomorphine (APO)^14^,^15^,^16^. We first treated mice with APO and then sacrificed them one hour after treatment and harvested the brain samples from the striatum for *in vivo* biotin-switch assay. We found that treatment of mice with APO resulted in an increased TH S-nitrosylation in the striatum as shown by the biotin-switch assay (Fig. 5A). To determine if this increased S-nitrosylation would affect the TH enzymatic activity, we performed TH real-time enzymatic kinetic assay from the striatum tissue lysate of control or APO treated mice. In agreement with the biotin-switch assay, treatment of APO significantly increased the TH enzymatic activity in the striatum (Fig. 5B). To further confirm this increased TH enzymatic activity was caused by S-nitrosylation, we treated the striatum samples isolated from mice after APO treatment with DTT to reverse the S-nitrosylation modification in TH. We found that treatment of the striatum samples with DTT completely abrogated the APO induced increase in TH enzymatic activity (Fig. 5C), further confirming that the APO induced enhancement of TH enzymatic activity was through TH S-nitrosylation and was reversible. To determine if specific type of dopaminergic receptor was involved in the enhancement of TH enzymatic activity by S-nitrosylation, we performed similar experiments with the DA receptor D1 agonist SKF-82958 or DA receptor D2 agonist quinpirole with paradigms as in previous studies^17^,^18^. We found that treatment of mice with SKF-82958 or quinpirole resulted in increased TH S-nitrosylation in the striatum as shown by the biotin-switch assay (Fig. 6A). To determine if this increase in TH S-nitrosylation would affect the TH enzymatic activity, we performed TH real-time enzymatic kinetic assay from the striatum tissue lysate of control, SKF-82958 or quinpirole treated mice. In agreement with the biotin-switch assay, treatment of both SKF-82958 and quinpirole significantly increased the TH enzymatic activity in the striatum (Fig. 6B). These results suggested that activation of both DA receptor D1 and D2 was involved in the enhancement of TH enzymatic activity through S-nitrosylation *in vivo*.

## Discussion

In this study, we found that TH can be S-nitrosylated both *in vitro* and *in vivo* and this modification is reversible (Figs 1 and 5). We identified the site of TH S-nitrosylation at cys 279 and this modification enhances the enzymatic activity of TH (Figs 2, 3 and 4). We further found that TH S-nitrosylation enhance its enzymatic activity through increasing the V_max_ without affecting the K_m_ (Fig. 3). To determine if this phenomenon can be observed *in vivo* in the dopaminergic system, we first treated mice with the non-selective DA agonist apomorphine to activate the dopaminergic transmission to see if this can enhance the TH S-nitrosylation and also its enzymatic activity. In agreement with our *in vitro* results, treatment of apomorphine can increase TH S-nitrosylation and also its enzymatic activity (Fig. 5), suggesting that NO can modulate TH’s enzymatic activity *in vivo* through S-nitrosylation in the dopaminergic transmission. To further determine if specific DA receptor is involved in this phenomenon, we treated mice with DA D1 (SKF-82958) and D2 (quinpirole) receptor agonists to see if this can also enhance the TH S-nitrosylation and also its enzymatic activity. We found that both activation of DA D1 and D2 receptors contributes to increasing TH S-nitrosylation and also its enzymatic activity (Fig. 6). In addition, we had also roughly estimated what was the percentage of TH S-nitrosylation both *in vitro* and *in vivo* in this study and found that they were at around 24% and 15% range (Fig. S1).

These results suggest a novel mechanism in which nitric oxide through TH S-nitrosylation can regulate TH’s enzymatic activity during dopaminergic transmission *in vivo*. NO signaling is known to modulate dopaminergic transmission in different DA circuitry and neuronal nitric oxide synthase (nNOS) has been found in some neurons in the striatum^19^,^20^,^21^. Possible mechanisms have been shown to involve modulation of DA transporter activity and activation of soluble guanylyl cyclase by NO^19^,^20^. Some other studies have also found that NO donors can enhance frequency dependent release of DA^15^,^22^, suggesting a facilitating mechanism of dopaminergic transmission by NO. In this study, we found a novel mechanism of how NO can cross talk with the dopaminergic system via the modulation of TH’s enzymatic activity through S-nitrosylation. Studies have found that TH’s enzymatic activity can be regulated by phosphorylation of the N-terminal regulatory domain of TH^2^,^8^. It is interesting to note that in this study, we found that S-nitrosylation at more C-terminus region can enhance TH’s enzymatic activity, suggesting multiple levels of TH PTM can regulate its activity through different regions via different mechanisms. Previous study have also shown that peroxynitrite and aged GSNO can induce S-thiolation in TH and inhibit its enzymatic activity, suggesting that NO species can induce a number of cysteine residue modifications in TH and regulate its enzymatic activity^23^. TH can also be modified by NO through tyrosine nitration and this modification inactivates its enzymatic activity^2^,^7^. This inactivation of TH by tyrosine nitration has been shown to increase in animal model of Parkinson’s disease (PD)^24^. PD is a neurodegenerative movement disorder that is marked by the selective degeneration of dopaminergic neurons in the substantia nigra^25^,^26^,^27^. The cause of the specific neurodegeneration is not completely understood but many factors have been suggested including aging and increase in nitrosative stress in the brain^25^,^28^. It is tempting to speculate that dysfunction of the NO mediated facilitation of DA transmission cause the increase in nitrosative stress and contribute to the pathogenesis of PD during aging. However this speculation will require a more detail study and understanding of the relationship between NO signaling and dopaminergic neurotransmission in the brain. In conclusion, our study show a novel mechanism of how NO through S-nitrosylation of TH can enhance the rate-limiting step in the production of dopamine.

## Materials and Methods

### Chemicals and plasmids

All chemicals were purchased from Sigma-Aldrich (St. Louis, MO) unless otherwise stated. TH construct was bought from Source Bioscience (Santa Fe Springs, CA), and then was sub-cloned into pCMV-Tag2B vector for cell culture study and into pET28a vector and pGEX4T-1 vector for production of recombinant protein. TH mutants C207H and C279H were made by polymerase chain reactions (PCR) mutagenesis using wild-type TH construct as template and cloned into pCMV-Tag2B vector. All constructs were verified by DNA sequencing.

### Recombinant Protein Purification and Antibodies

Recombinant proteins with His or GST tag were expressed in Rosetta (DE3) pLys *Escherichia coli* (Novagen, Bilerica, MA). Cultures were grown until linear phase (0.6 OD) and expression of proteins were induced by 250 μM IPTG at 18 °C for 20 h. As iron is essential for TH activity, to maintain TH activity 2% glucose and 1 mM FeSO_4_ together were added when inducing the expression of recombinant TH as reported^29^. The culture was then harvested and recombinant proteins were purified by Ni^2+^-nitrilotriacetic acid resin (Qiagen) or GSH-Sepharose (GE Healthcare, Piscataway, NJ) according to the manufacturer’s protocol, respectively. The obtained proteins were then dialyzed in PBS.

For the generation of anti-TH antibody, purified his-tagged TH recombinant protein in PBS was mixed with equal volume of Freund’s adjuvant forming an emulsion. The mixture was then used to immunize a New Zealand white rabbit and the Anti-TH antibody was purified from serum with GST-tagged TH conjugated to CNBr activated Sepharose affinity purification (GE Healthcare, Piscataway, NJ). The specificity of the antibodies was verified by Western blot analysis.

### Cell culture and transfection

HEK293T cells were cultured in DMEM plus 10% Fetal Bovine Serum and 1% Penicillin Streptomycin solution (Gibco Thermo Fisher Scientific, Waltham, MA). PC12 cells were maintained in DMEM plus 10% Horse Serum, 5% Fetal Bovine Serum and 1% Penicillin Streptomycin solution. Both cell lines are cultured at 37 °C with 5% CO_2_. DNA plasmids were transfected into cells with Lipofectamine™ LTX and Plus Reagent (Invitrogen, Thermo Fisher Scientific, Waltham, MA) according to manufacturer’s protocol.

### *In vitro* and *in vivo* biotin-switch assay

S-nitrosoglutathione (GSNO) was prepared as described before^30^. Glutathione (GSH, 100 mM) was reacted with a same molar of NaNO_2_ in acidic condition (0.075 M HCl) for 10 min at room temperature in darkness. Then same molar of NaOH was added to neutralize the mixture and GSNO was precipitated by pre-chilled acetone at −20 °C, and then washed with pre-chilled (−20 °C) acetone for 3 times. Precipitated GSNO was then dissolved in MilliQ water and the concentration of GSNO is determined by spectroscopy at the wavelength of 334 nm with the extinction coefficients as described (ε_334_ = 900 M^−1^ cm^−1^)^30^. GSNO was freshly prepared at the day of each experiment.

For *in vitro* S-nitrosylation assay, cell lysates in HENT buffer (250 mM Hepes, 1 mM EDTA, 0.1 mM Neocupoine, 1% Triton X-100, 1 mM aprotinin, 1 mM leuprptin, 1 mM benzamidine, 10 mM PMSF, pH 7.7) from HEK293T cells transfected with WT or mutant TH, or PC12 cells were treated with GSNO/GSH or NOC5 (Calibiochem, Millipore, Temecula, CA) for 30 min. Samples were then passed through G25 Sephadex desalting spin column (GE Healthcare, Piscataway, NJ) to remove the NO donors and incubated with 10 mM methanethiosulfonate (MMTS) (Thermo-Fisher, Waltham, MA) at 50 °C water bath for 20 min with 2% SDS. Excess MMTS in the samples were removed by G25 Sephadex desalting spin column and the samples were then incubated with 0.4 mM Biotin-HPDP (Thermo-Fisher, Waltham, MA) and 5 mM ascorbate at cold room temperature overnight with rotation. Excessive biotin-HPDP was then removed by acetone precipitation. Precipitated protein was then pelleted by centrifugation and re-suspended in 400 μl of HENS buffer (250 mM Hepes pH7.4, 1 mM EDTA, 0.1 mM Neocupoine, 1% SDS). To detect the overall amount of S-nitrosylated protein in the samples, 50 μl of samples were aliquoted and mixed with SDS-PAGE buffer without 2-mercaptoethanol for Western blot analysis using using Neurtravidin-HRP (Thermo-Fisher, Waltham, MA). The remaining samples were then diluted by 1 ml of neutralization buffer (20 mM Hepes pH 7.4,100 mM NaCl, 1 mM EDTA, 0.5% Triton X-100) and mixed with 20 ul Neutravidin-agarose bead (Thermo-Fisher, Waltham, MA). After incubating at 4 °C for 2 h, the Neutravidin-agarose bead was then pelleted by centrifugation and washed five times with neutralization buffer (20 mM Hepes pH 8.0, 100 mM NaCl, 1 mM EDTA, 0.5% Triton X-100) with 0.6 M NaCl and once with TBS. Samples were then eluted by reducing SDS-PAGE sample buffer and analyzed by Western blot.

For *in vivo* analysis of mouse tissues, brain lysates were homogenized in HENT buffer with MMTS and then followed the same protocol as in the *in vitro* biotin-assay. Ascorbate or biotin-HPDP was omitted to serve as control. In addition, pretreatment of either 200 μM DTT or 200 μM HgCl_2_ was served as controls in reversing the endogenous S-nitrosylation.

### Fluorometric detection of S-nitrosylated TH

Fluorometric assay using 2,3-diaminonaphthalene (DAN) was performed as described^30^. In brief, purified his-tagged TH was treated with 100 μM DTT (Dithiothreitol) for 30 min and passed through G25 Sephadex desalting spin column once. Afterwards the samples were treated with 250 μM of GSH or GSNO for 30 min and then passed through G25 Sephadex desalting spin column twice. The samples were then incubated with 100 μM HgCl_2_ and 100 μM 2,3-diaminonaphthalene (DAN) for 1 h. The fluorescence generated by the reaction product 2,3-napththyltrazole (NAT) was measured at an excitation wavelength of 355 nm and an emission wavelength of 460 nm.

### Real time TH enzymatic kinetic assay

TH enzymatic kinetic assay was performed as described^11^. The assay is based on the formation of chromopore dopachrome by the reaction between L-DOPA produced by TH and sodium periodate (NaIO_4_). Reaction buffer containing 100 mM HEPES (pH 6.8), 200 μM L-tyrosine, 500 μM FeSO_4_, and 0.25 mM BH_4_ was prepared. NaIO_4_ (200 μM) in water was prepared as the substrate to react with L-DOPA and 100 μl was first aliquot to a 96 well plate. Samples with 20 μg of recombinant protein, cell or brain lysates in 20 μl of PBS were mixed with 80 μl of reaction buffer to form the reaction mixture. After mixing, 100 μl of reaction mixtures were added to the 96 well-plate with NaIO_4_ and the formation of dopachrome was monitored by colorimetric reader that recorded the absorbance of samples at 475 nm every 20 s for 10 min at 37 °C.

### V_max_ and K_m_ determination

V_max_ and K_m_ determination is based on the Michaelis-Menten model with the equation V_0_ = V_max_ [S]/(K_m_ + [S]) in which V_0_ is the initial rate at the linear phase of the enzymatic reaction and [S] is the substrate concentration^12^,^13^. To determine the V_max_ and K_m_ for TH to convert L-tyrosine to L-DOPA, the optimum time to measure V_0_ for different concentrations of substrate was first found out. In brief, a standard curve to measure concentration of L-DOPA was established by mixing 100 μl of L-DOPA (0–50 μM) with 100 μl NaIO_4_ (200 μM) and the dopachrome formed was measured by absorbance at 475 nm. Then, reaction mixture containing 10 mM HEPES (pH 6.8), 200 μM L-tyrosine, 500 μM FeSO_4_ and 0.2 μg/μl His-TH was prepared. The reaction was initiated by combining 180 μl of reaction mixture and 20 μl of BH4 (2.5 mM). At time point of 0, 1, 2, 3, 4, 6, 8 and 10 min, 100 μl of reaction samples were then combined with 100 μl NaIO_4_ and the formation of dopachrome in these samples were measured by absorbance at 475 nm. The amount of L-DOPA formed in these samples was then calculated with the standard curve and the optimum time to measure V_0_ was determined to be 2 min. To calculate V_max_ and K_m_ for TH, similar reaction mixtures were prepared except different L-tyrosine concentrations (0–90 μM) were added. The reaction was then initiated by combining 180 μl of reaction mixture and 20 μl of BH4 (2.5 mM). After two min, 100 μl of the reaction samples were mixed with 100 μl of NaIO_4_ and the formation of dopachrome in these samples were measured by absorbance at 475 nm. The amount of L-DOPA in these samples was calculated from the standard curve. V_0_ for different concentrations of L-tyrosine for TH was then calculated and V_max_ and K_m_ were determined with the non-linear regression function in GraphPad Prism software (GraphPad Software, San Diego, CA).

### Animals and treatment

All animals were housed, cared for, and experiments conducted according to relevant national and international guidelines. The animal protocols used have been reviewed and approved by the Animal Ethics Committee of The Hong Kong University of Science and Technology. All animal experiments were carried out by experienced personnel to minimize any discomfort or suffering of the animals. Three-month-old male wild-type C57BL/6 mice were injected (i.p.) with PBS as control, apomorphine at 5 mg/kg^14^,^15^,^16^, SKF-82958 hydrobromide at 2 mg/kg^17^ or (−)-Quinpirole hydrochloride at 2 mg/kg^18^. One hour after injection, mice were sacrificed by cervical dislocation and brain samples were harvested and biotin-switch or TH enzymatic assay was performed in these samples.

### Statistical analysis

Data are expressed as mean ± SEM. Significance was determined by ANOVA or Student’s *t* test. All statistical analyses were performed by GraphPad Prism software (GraphPad Software, San Diego, CA).

## Additional Information

**How to cite this article:** Wang, Y. *et al*. Novel enhancement mechanism of tyrosine hydroxylase enzymatic activity by nitric oxide through S-nitrosylation. *Sci. Rep.* **7**, 44154; doi: 10.1038/srep44154 (2017).

**Publisher's note:** Springer Nature remains neutral with regard to jurisdictional claims in published maps and institutional affiliations.

## Supplementary Material

Supplementary Information
