# Supplementary material for: Novel enhancement mechanism of tyrosine hydroxylase enzymatic activity by nitric oxide through S-nitrosylation
Source: Sci Rep. 2017 Mar 13;7:44154. doi: 10.1038/srep44154 (PMC5347018; doi:10.1038/srep44154)

Novel enhancement mechanism of tyrosine hydroxylase enzymatic activity by nitric oxide through S-nitrosylation

by

Yuanyuan Wang, Chun Chau Sung and Kenny K. K. Chung

Division of Life Science, State Key Laboratory of Molecular Neuroscience, The Hong Kong University of Science and Technology, Hong Kong, China.

Correspondence should be addressed to: Kenny K.K. Chung, Division of Life Science, The Hong Kong University of Science and Technology, Clear Water Bay, Hong Kong, China. Phone: + 852 2358 8019, Fax: + 852 2358 1552, Email: bckchung@ust.hk

Supplementary information

Fig. S1. Estimation of percentage of S-nitrosylation in *in vitro* and *in vivo* samples. In the *in vitro* (A) and *in vivo* (B) biotin switch assay, the supernatant before and after Neutravidin-agarose beads S-nitrosylated protein pull-down were analyzed by Western blotting. The density of the bands were quantified by Image J. The percentage of S-nitrosylated proteins were estimated by comparing the TH protein before and after pull-down. Figure S1 A-B included cropped images and the original blot images are included in the Supplementary Information file.

Table 1. The estimated percentage of TH S-nitrosylation *in vitro*

GSH	GSNO	GSNO	GSNO	GSNO	GSNO
200 μM	10 μM	50 μM	100 μM	200 μM	400 μM
7%	4%	16%	23%	24%	27 %

Table 2. The estimated percentage of TH S-nitrosylation *in vivo*

APO	APO
-	+
9%	15%

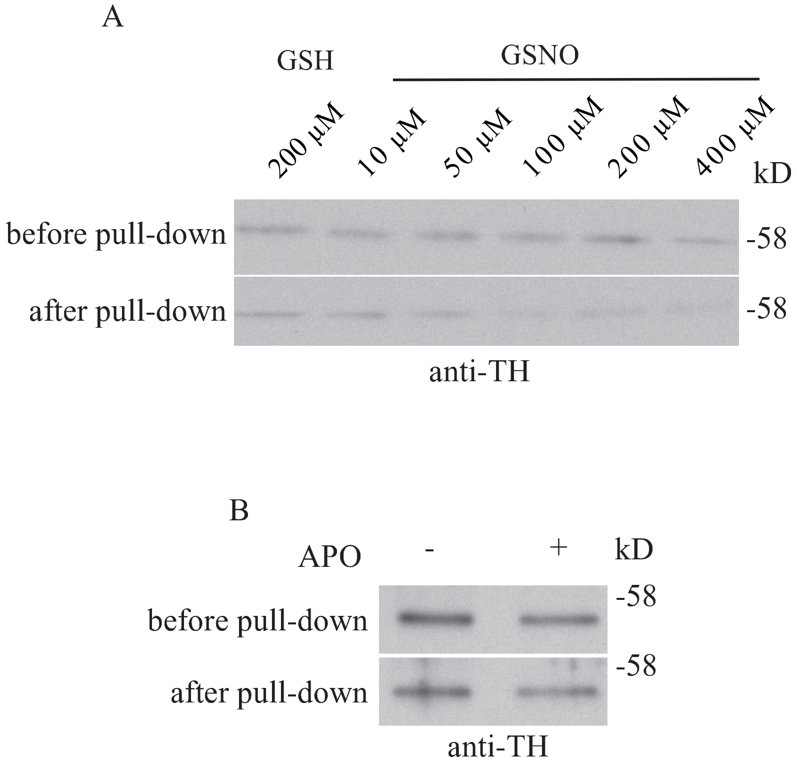
A
GSH
GSNO
200 µM
10 µM
50 µM
100 µM
200 µM
400 µM
kD
before pull-down
-58
after pull-down
-58
anti-TH
B
APO
-
+
kD
before pull-down
-58
after pull-down
-58
anti-TH

Fig S1

Original blot images of Figure 1 A-E, Figure 2 A-D, Fig 4 A-D, Figure 5 A-C, Figure 6 A&B and Figure S1 A&B

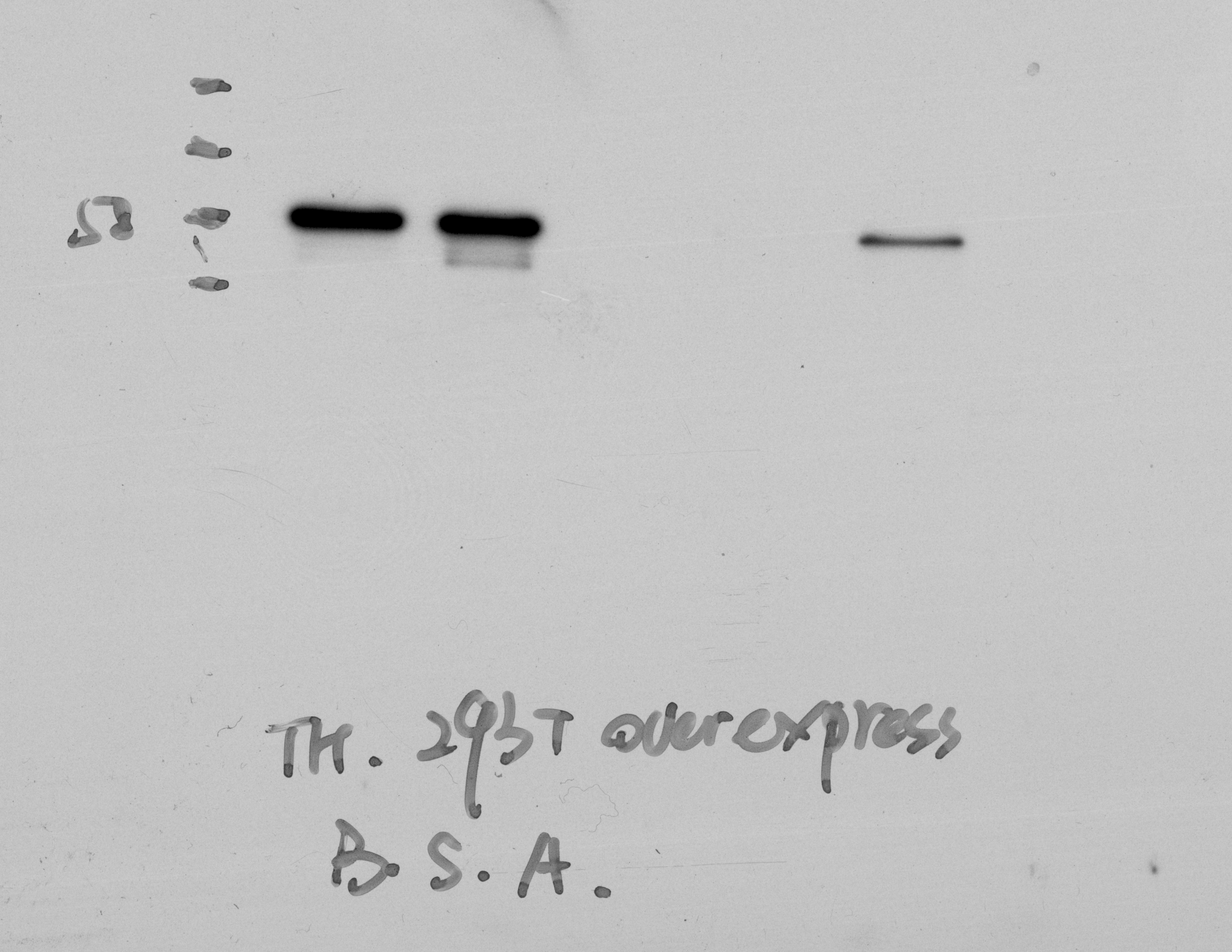
TH. 293T overexpress
B.S.A.

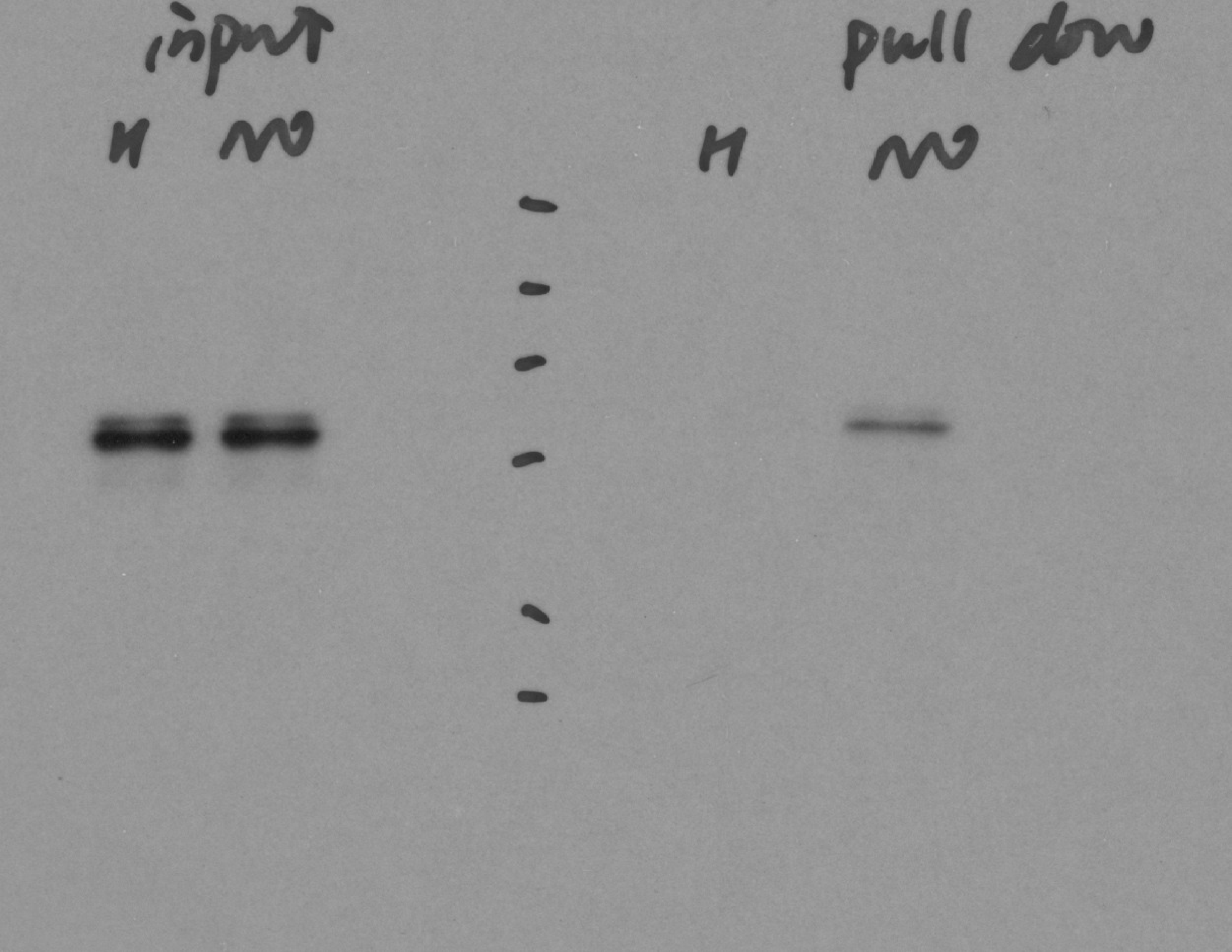
input
pull dow

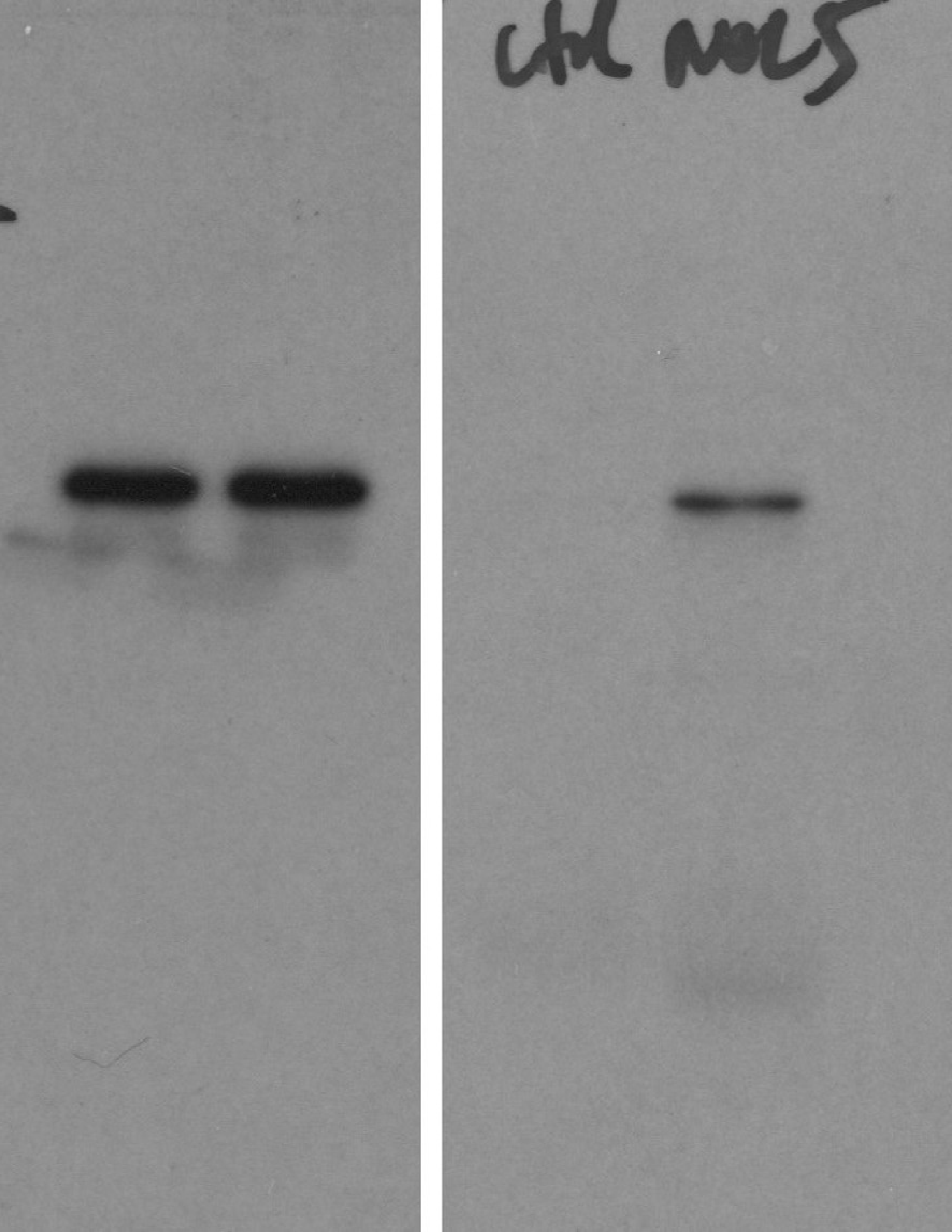

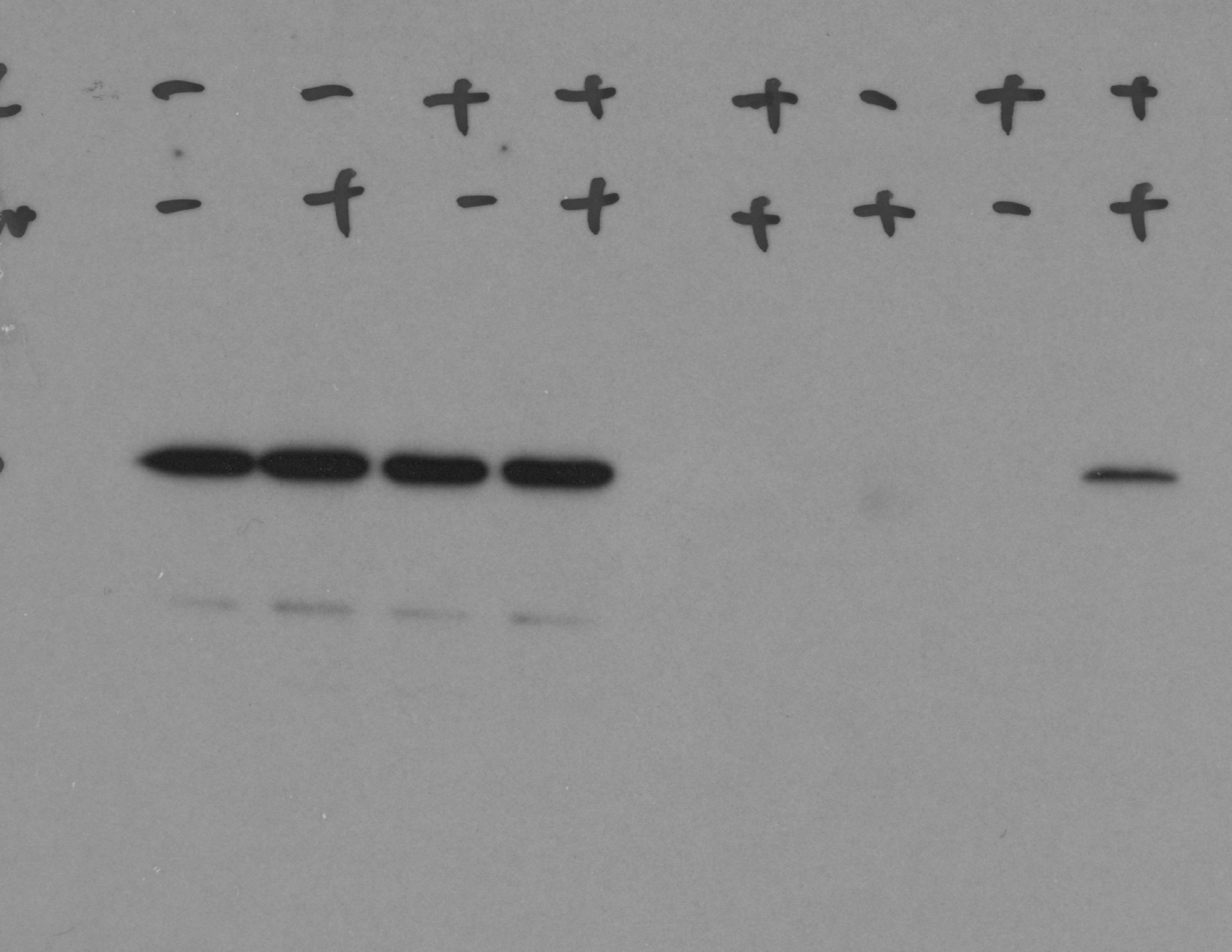

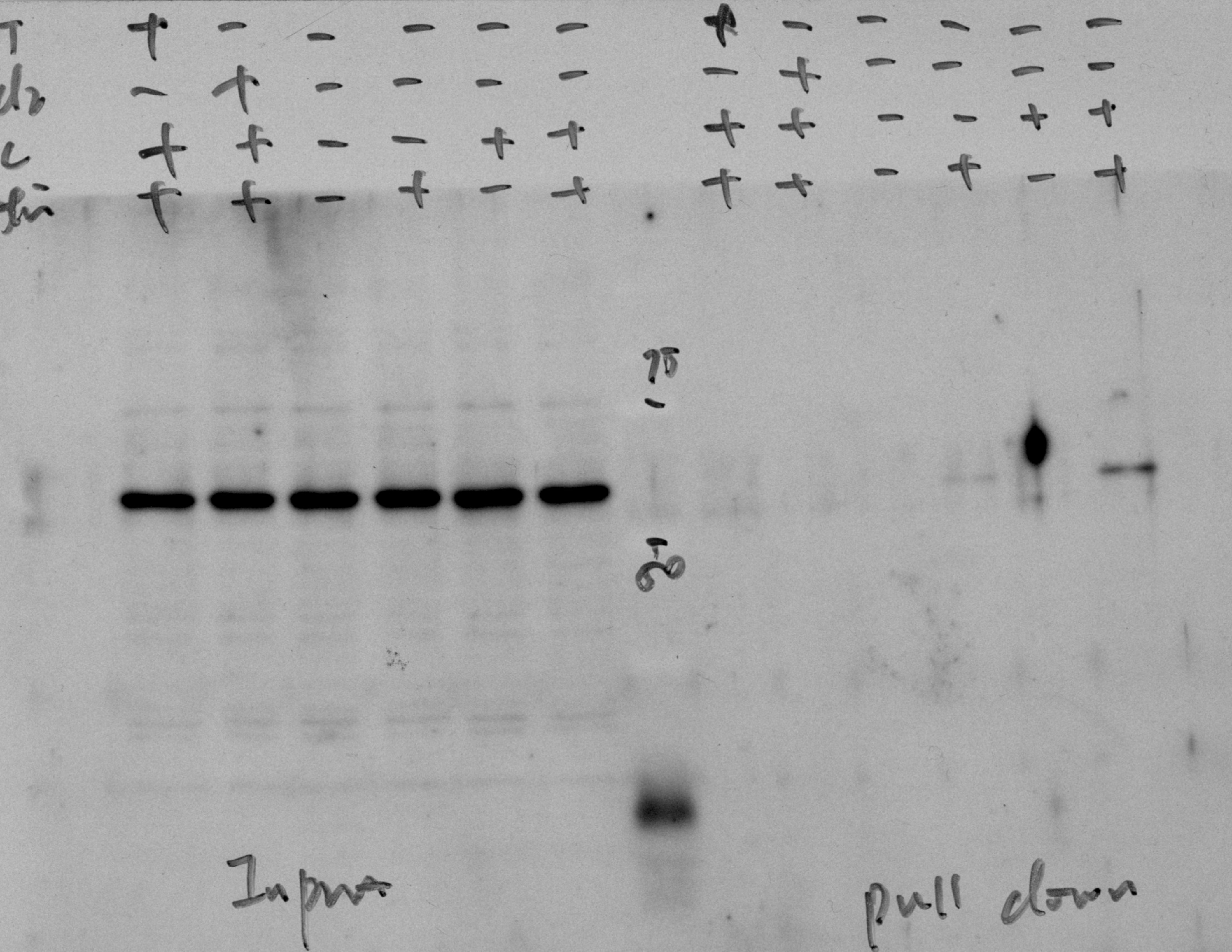
75
60
Input
pull down

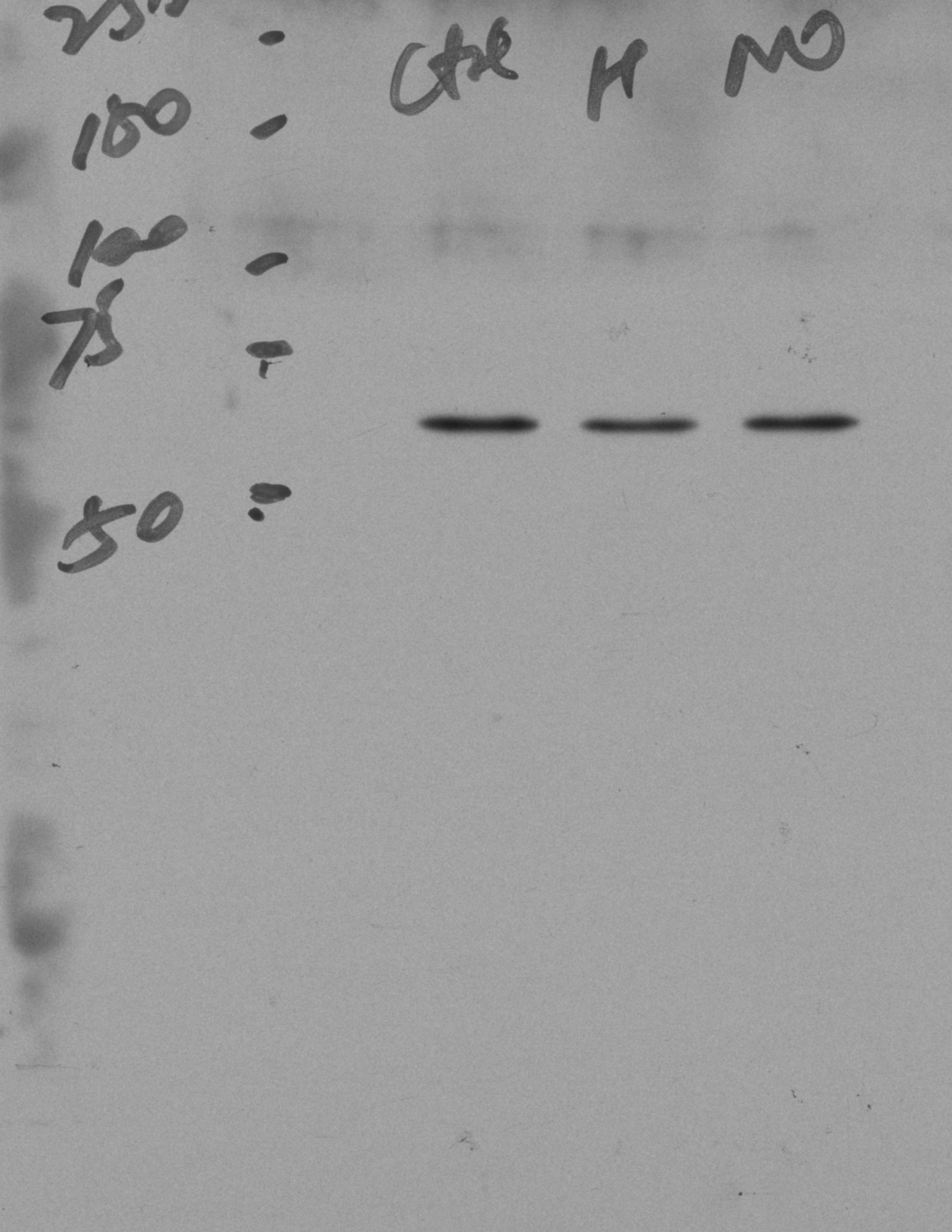
Ctrl
H
100
75
50

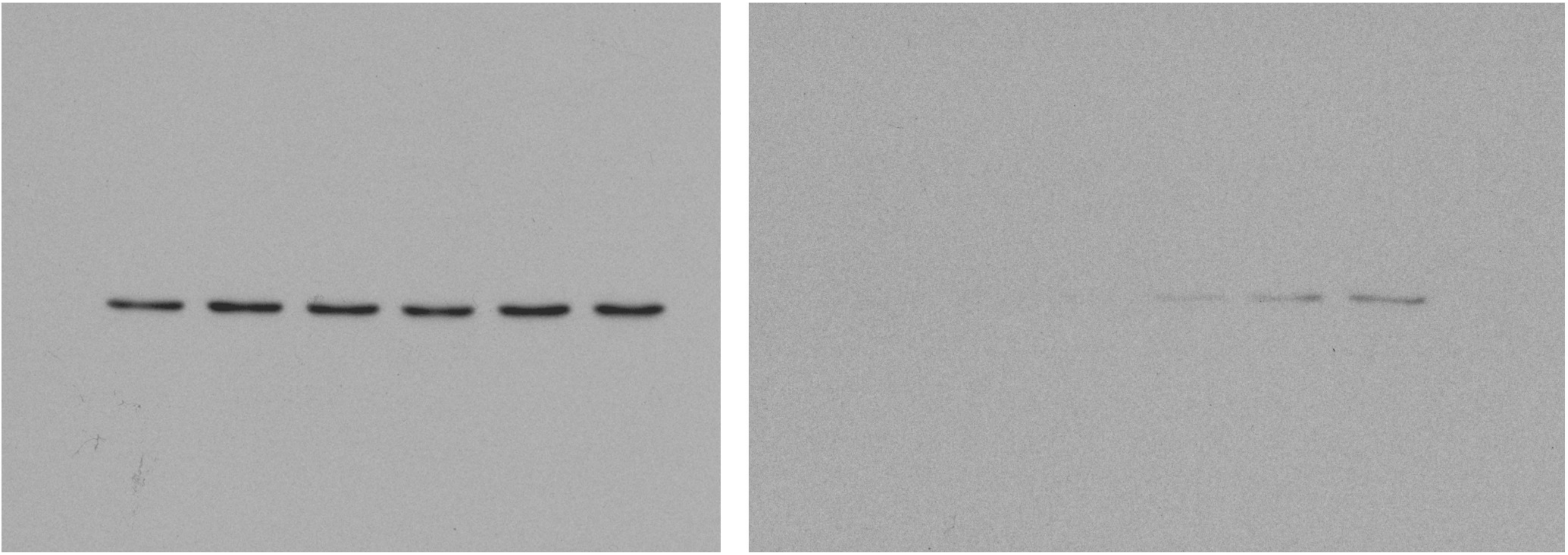

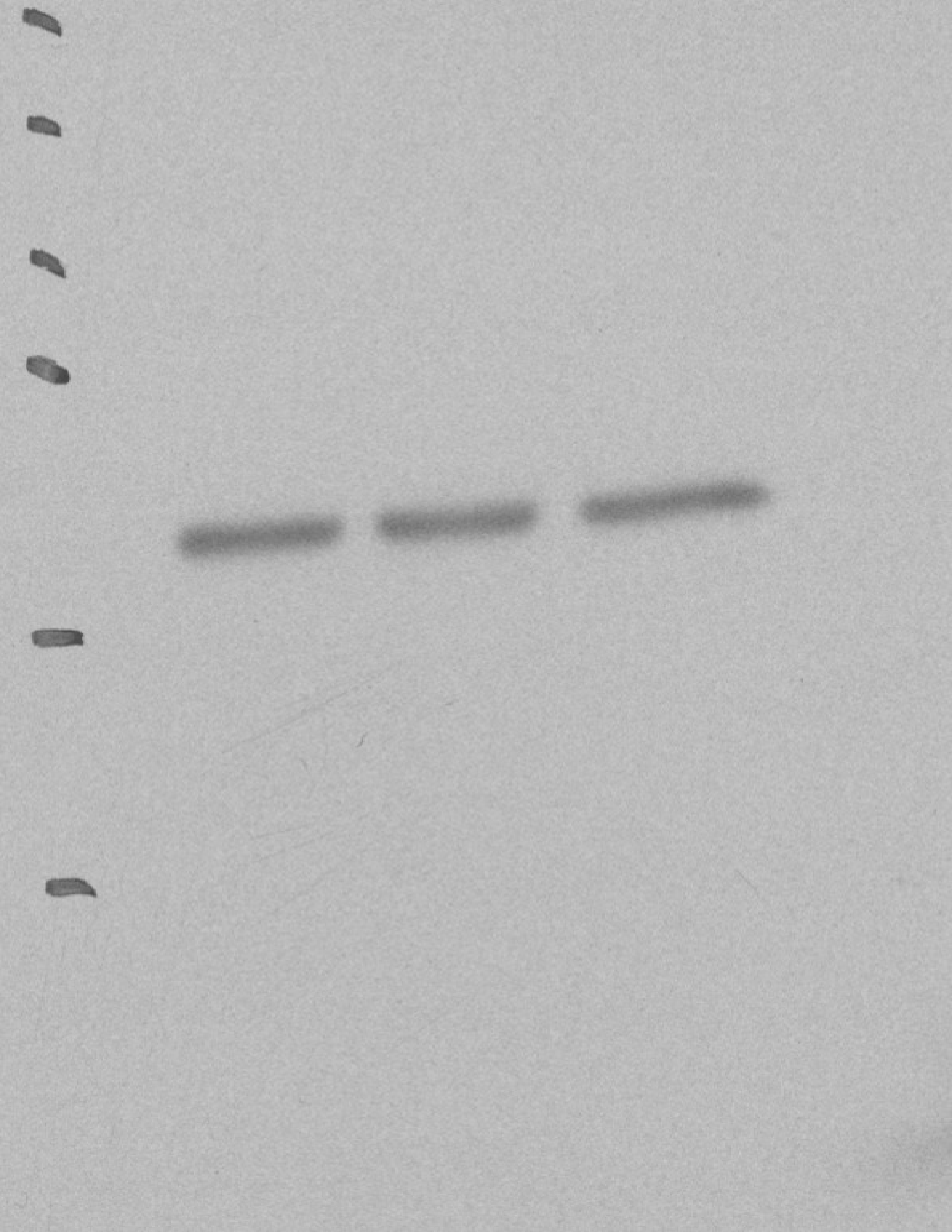

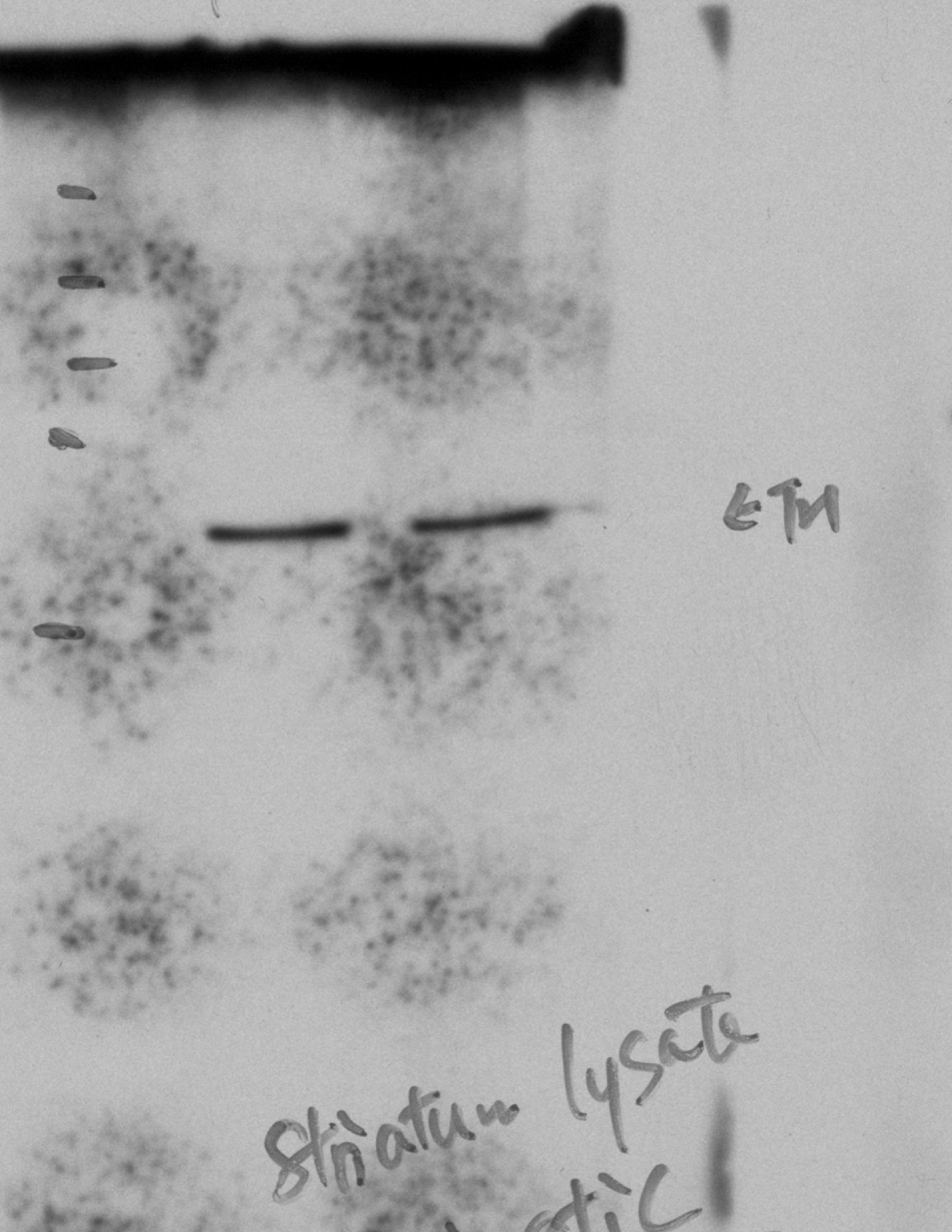
ETM
striatum lysate

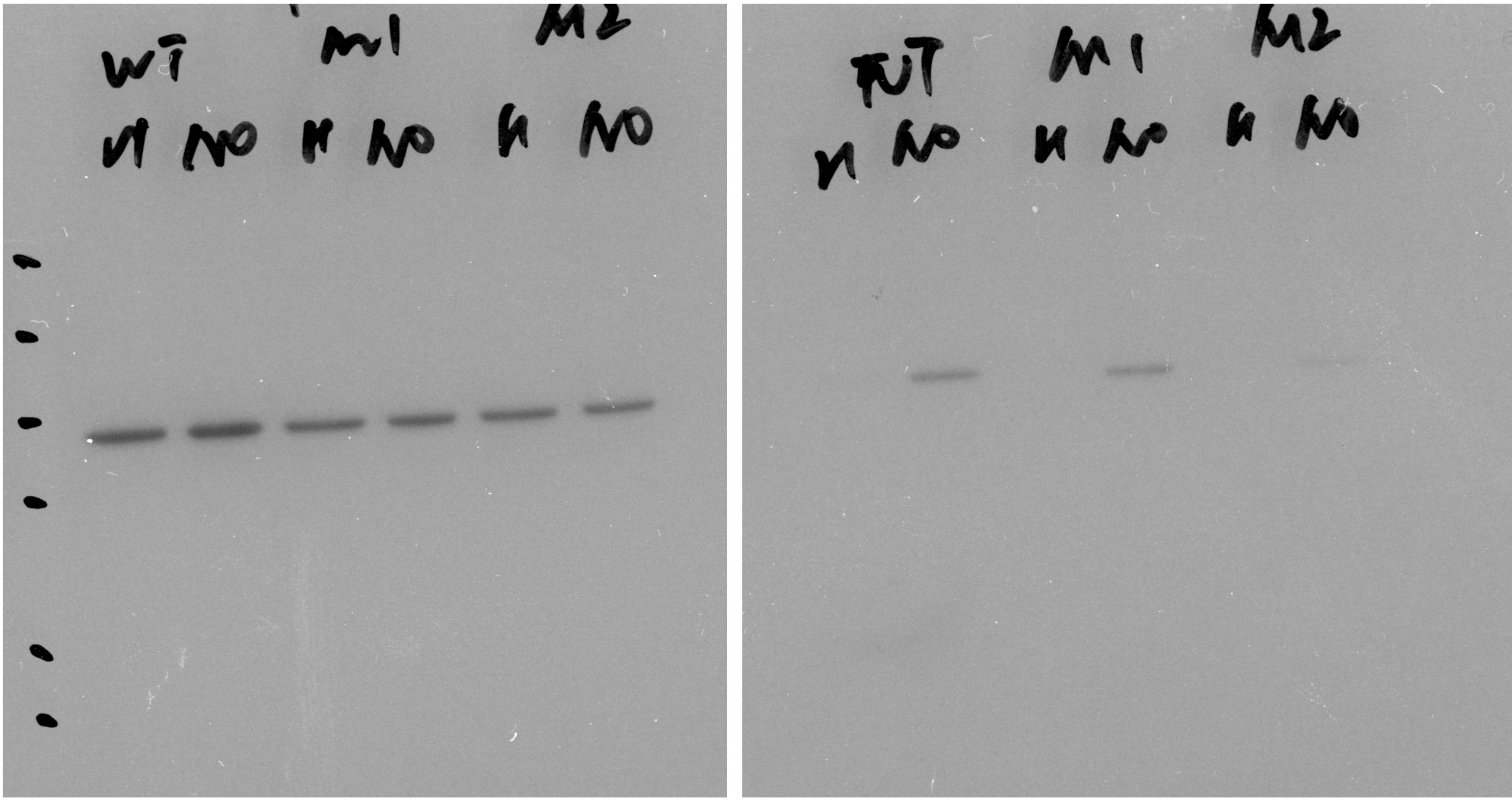
WT
M1
M2
H NO H NO H NO
WT
M1
M2
H NO H NO H NO

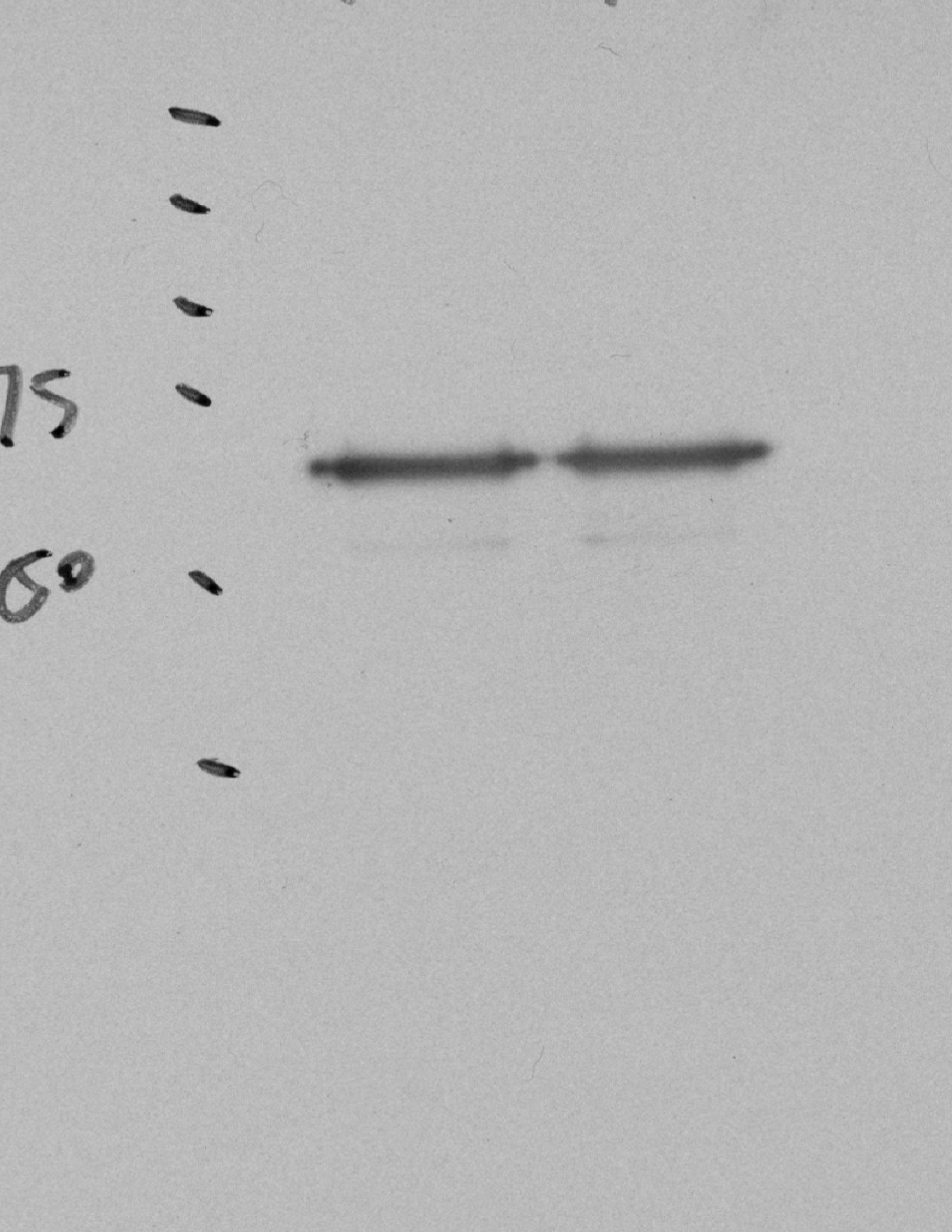
75
60

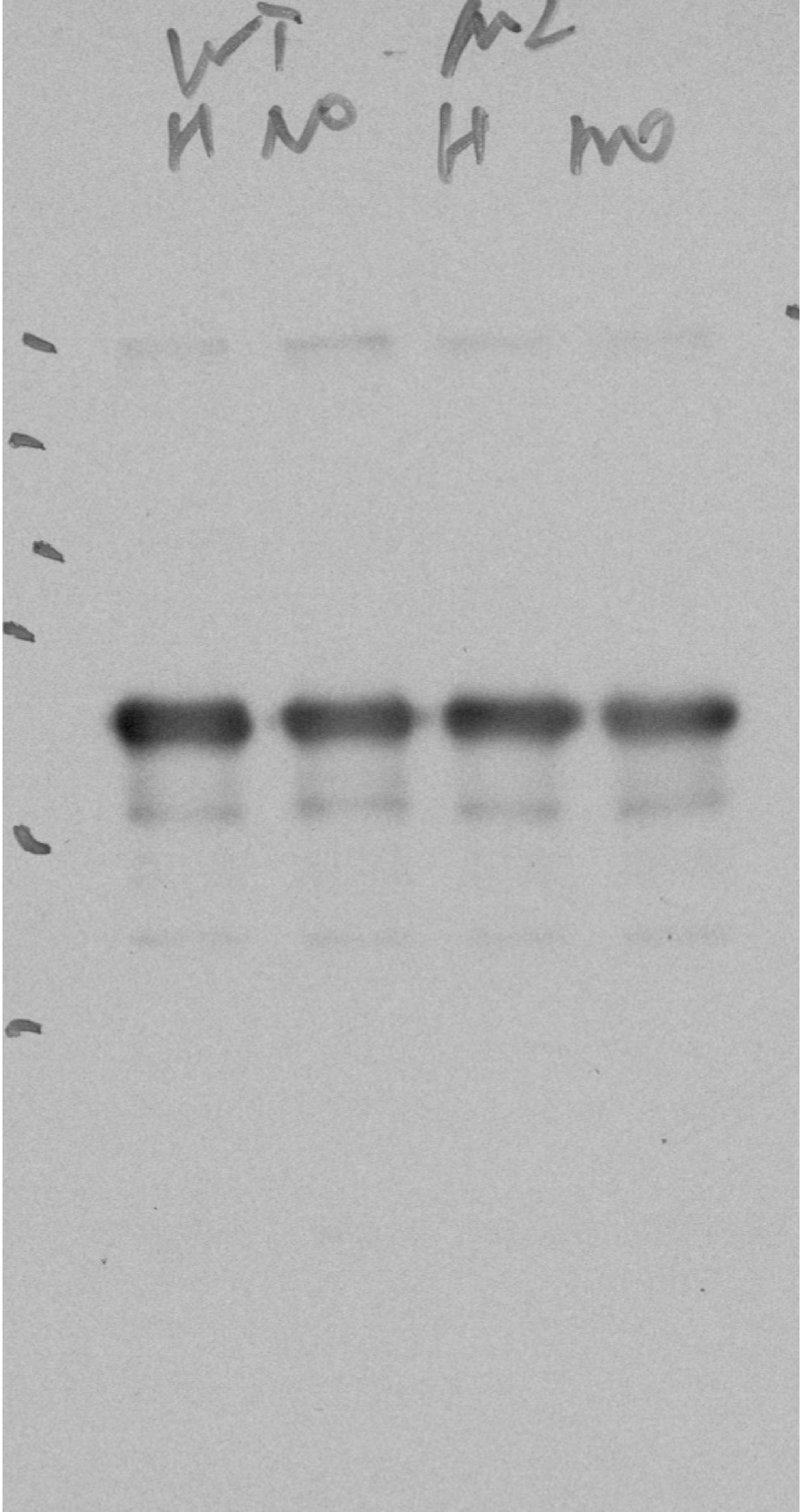
WT
M2
H NO
H mo

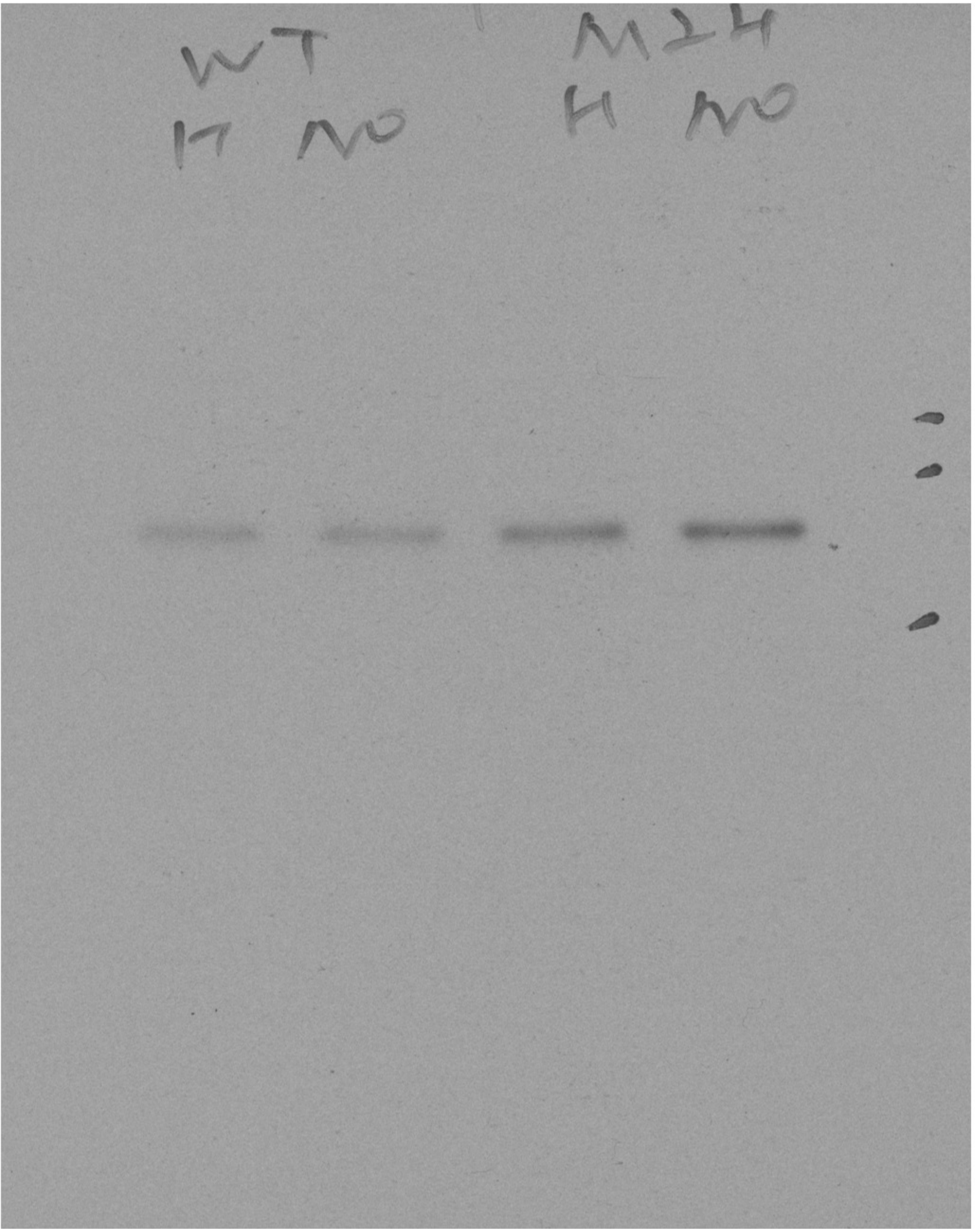
WT
H NO
M2H
H NO

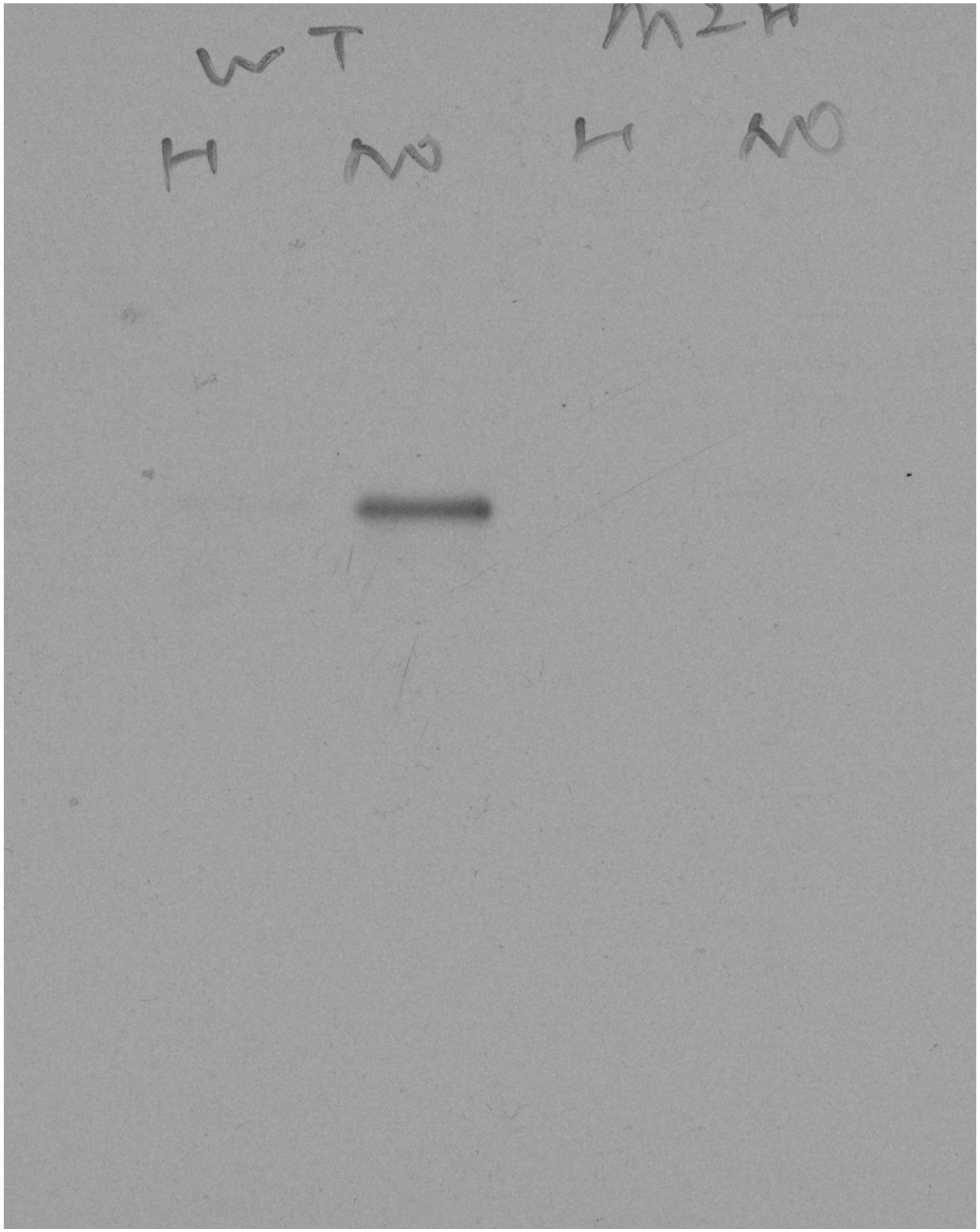
WT
H NO
M2H
H NO

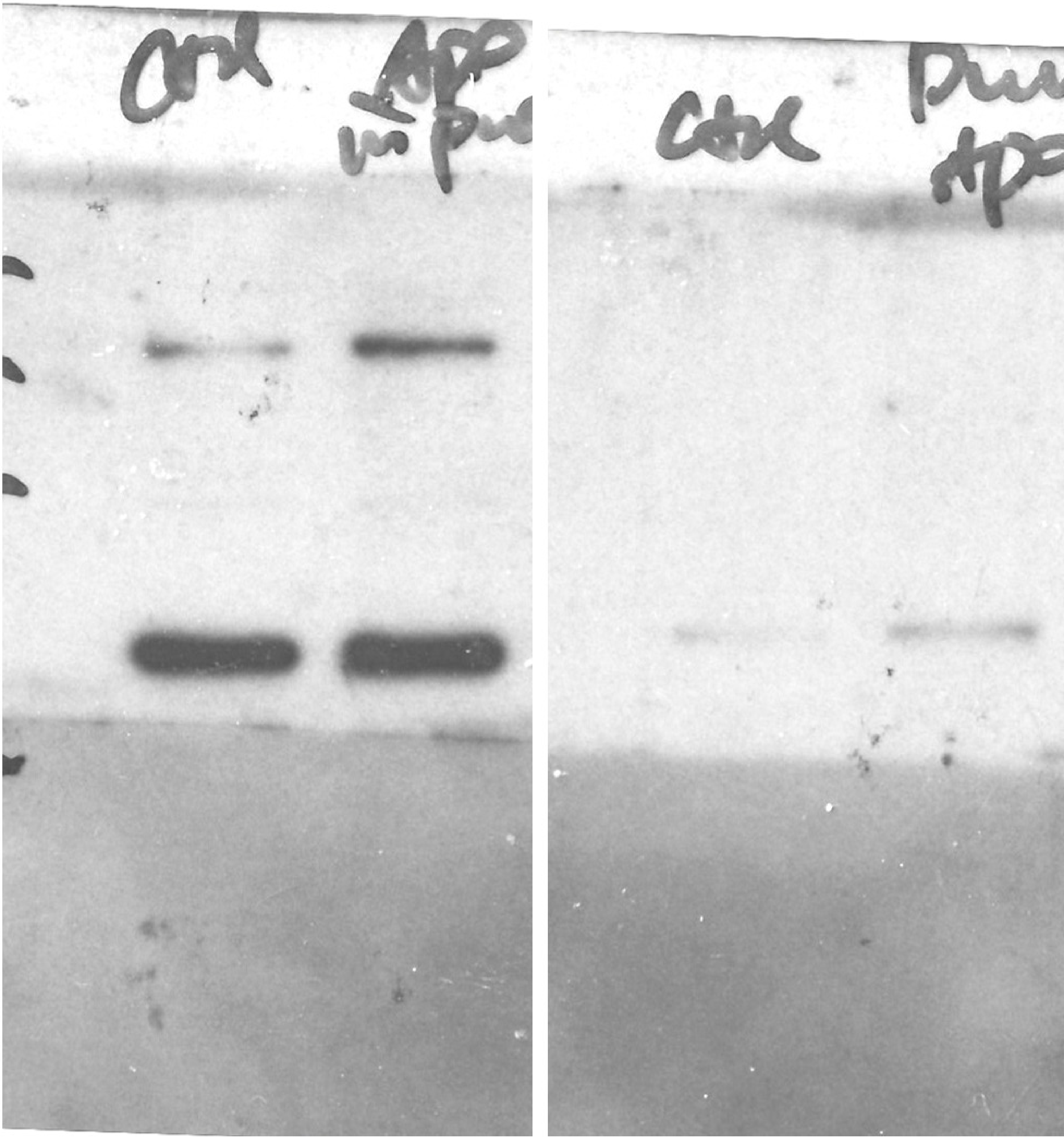
Ctrl
Ctrl

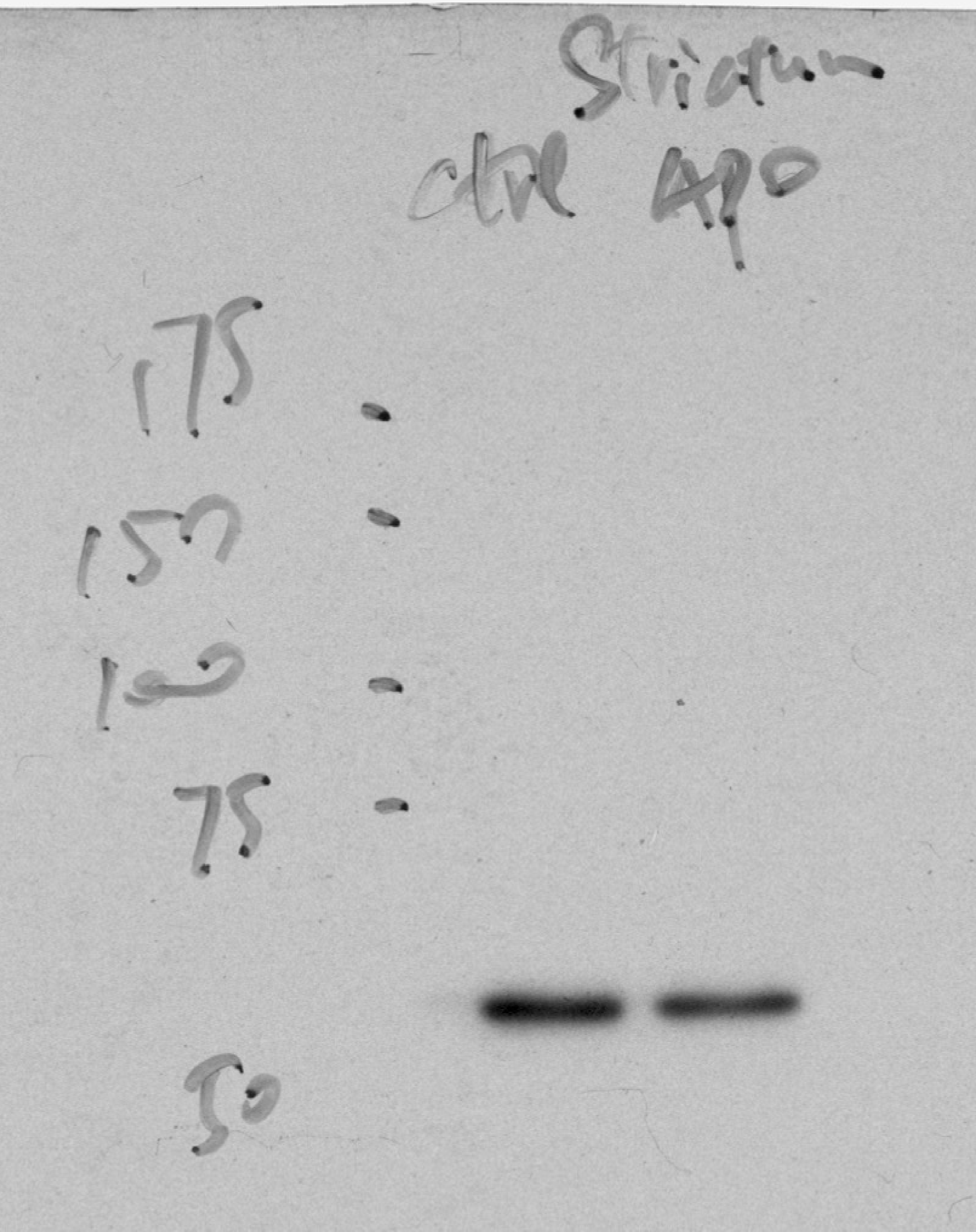
Striatum
ctrl
APO
75
50

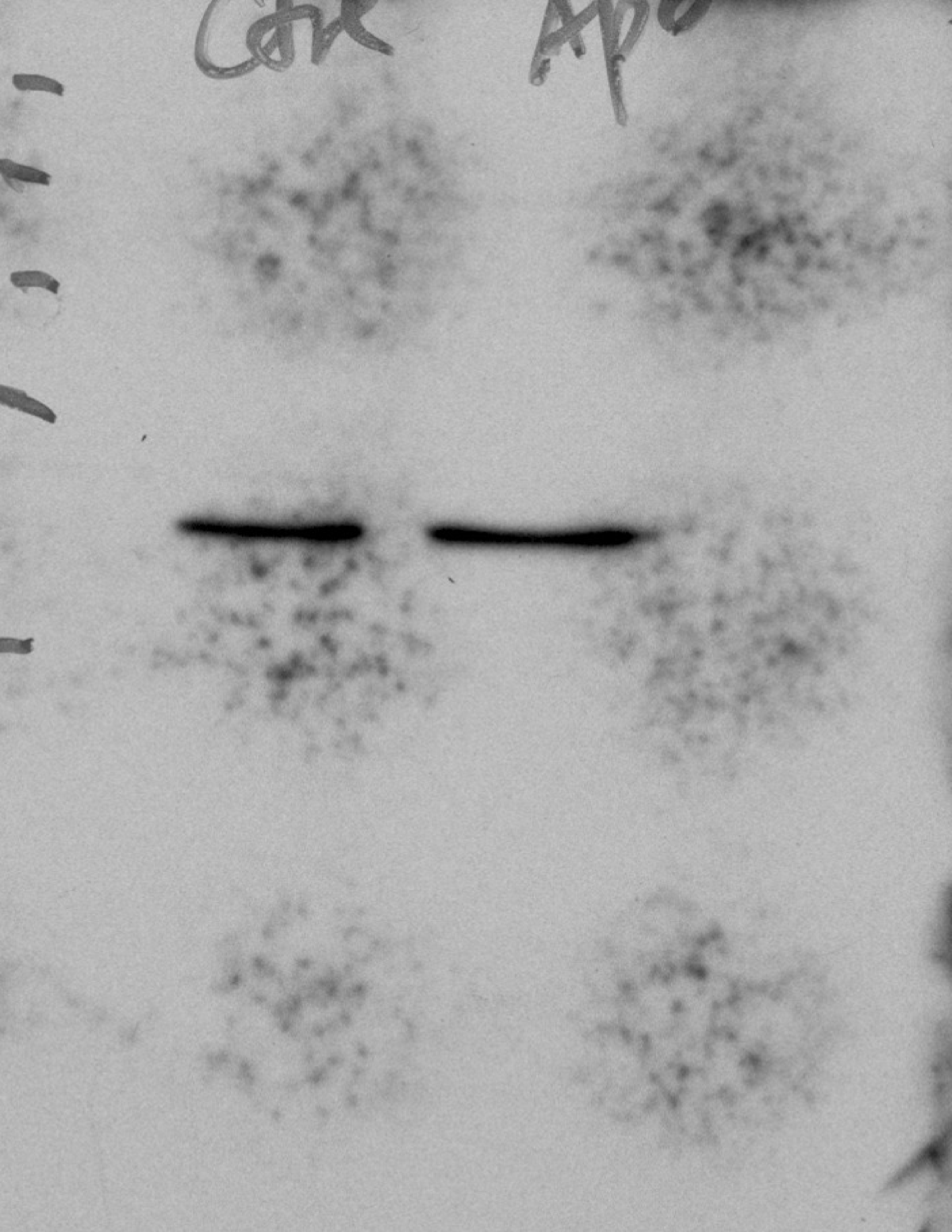

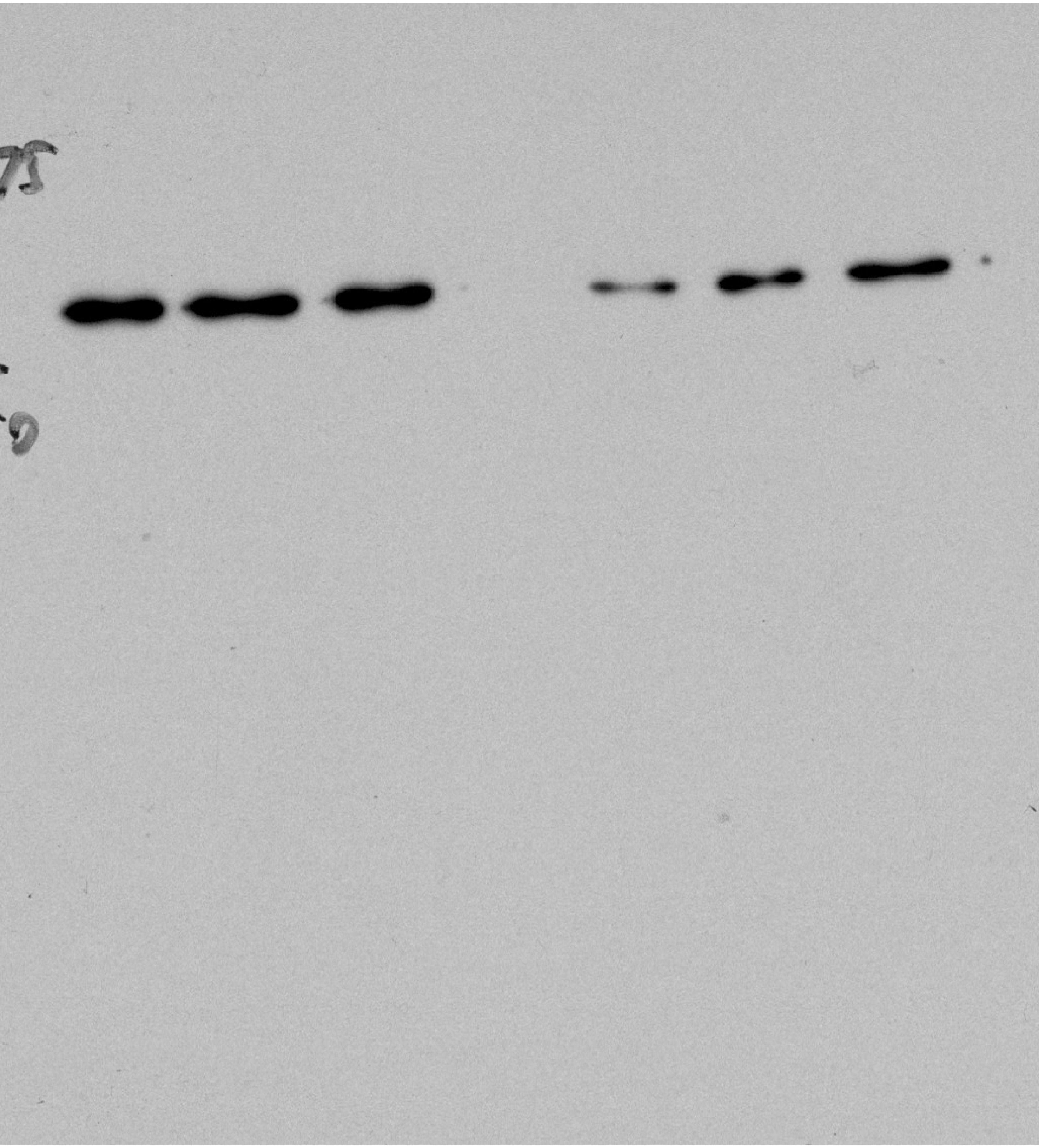
75

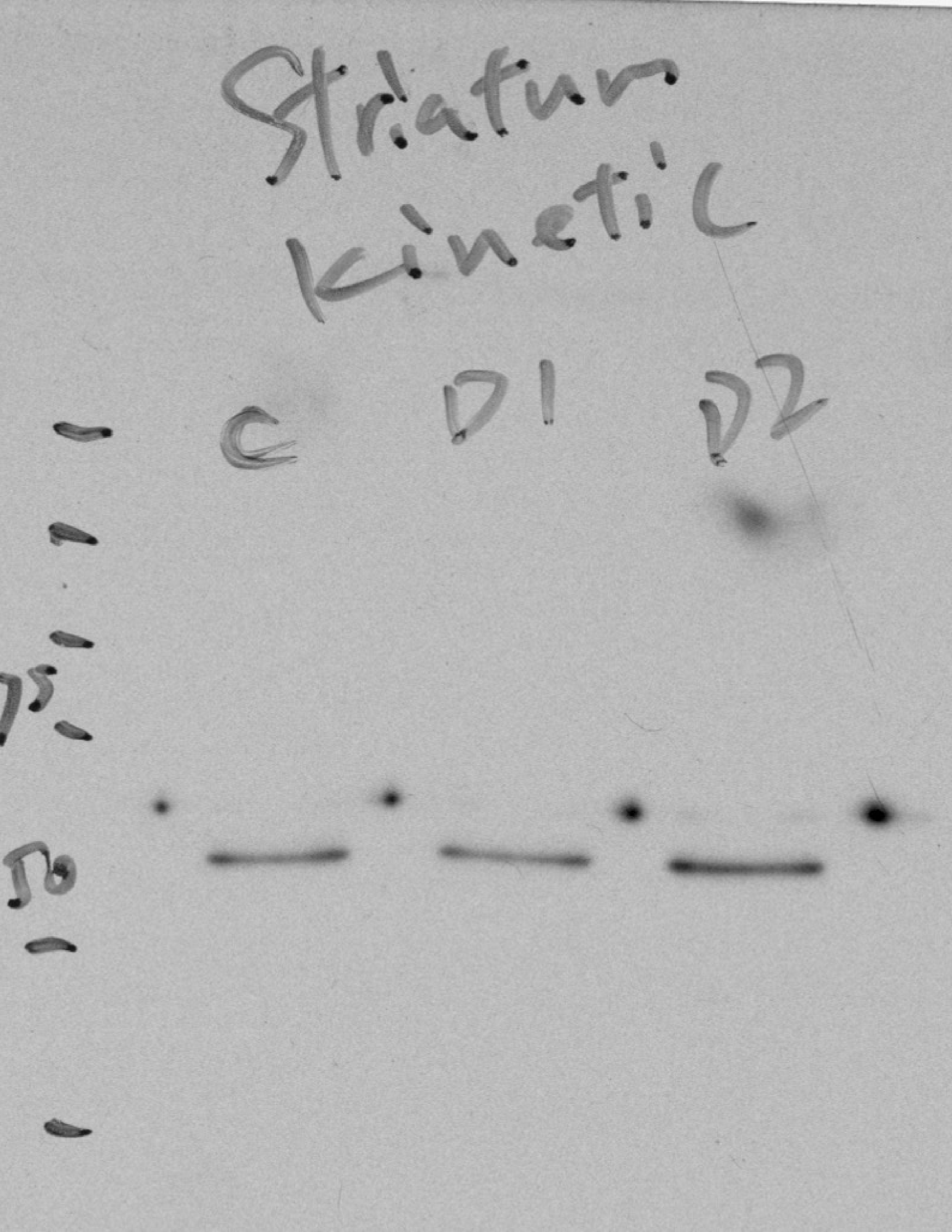
Striatum
kinetic
C
D1
D2
75
50

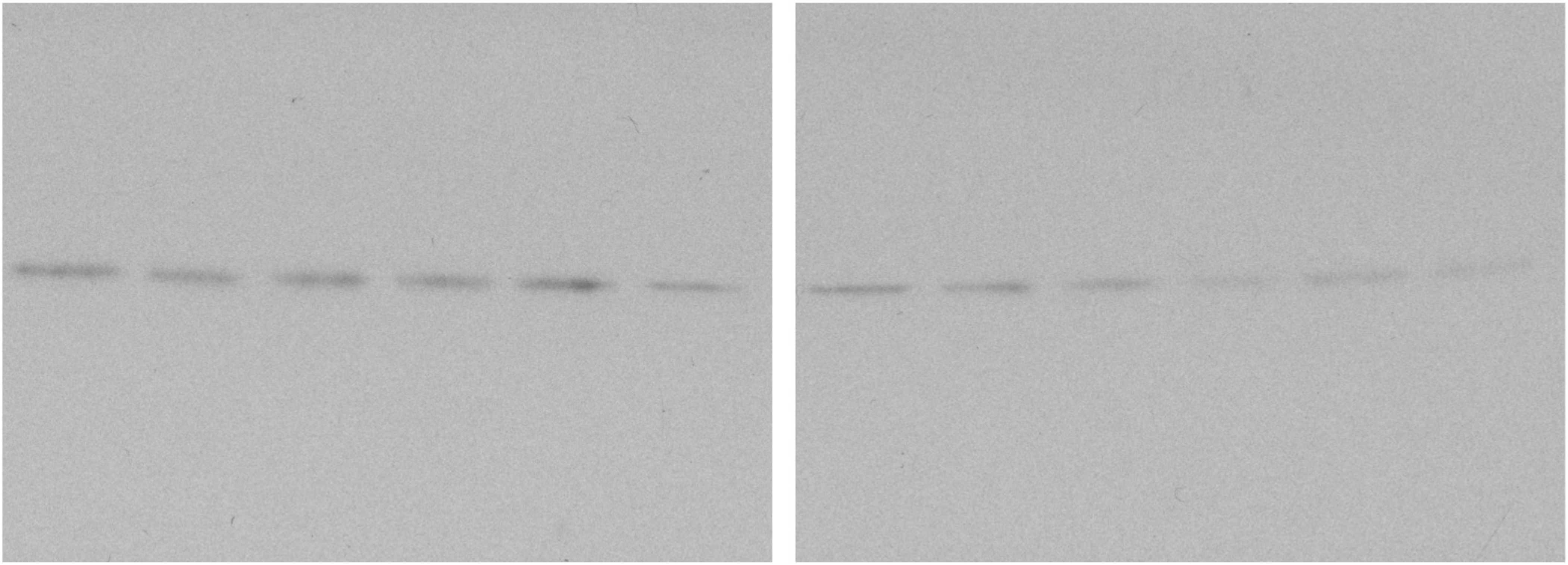

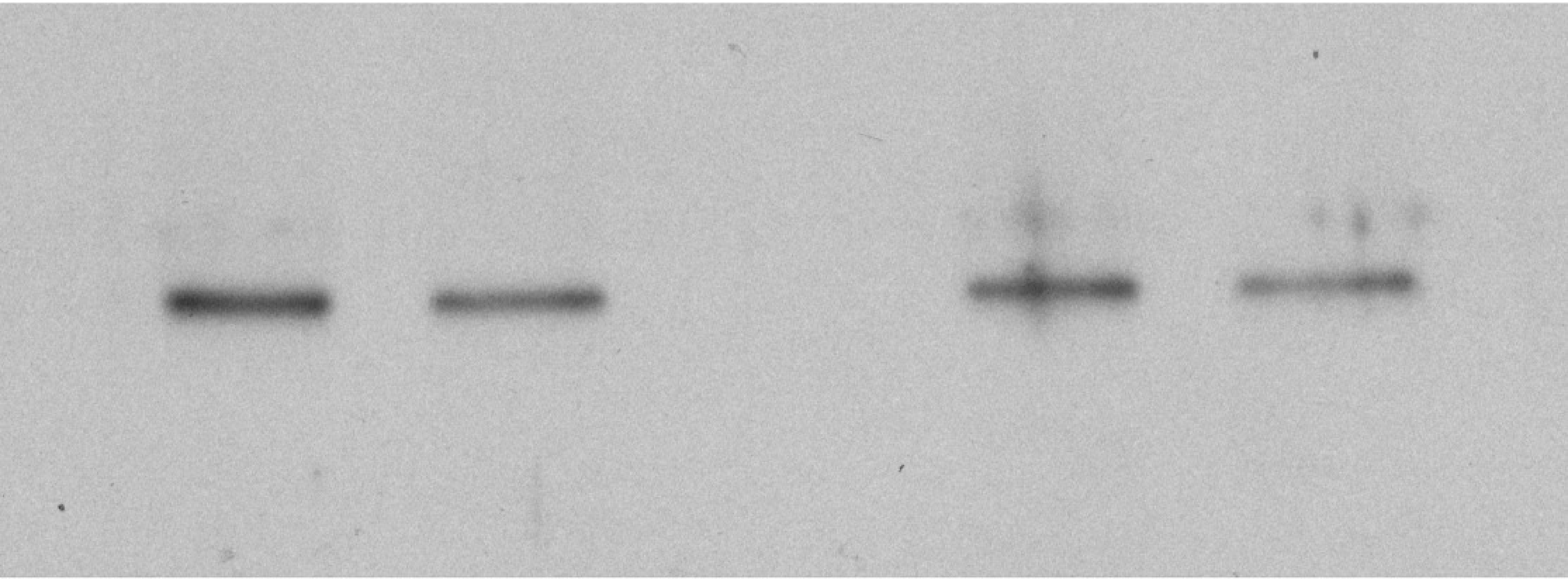

Supplement: Supplementary Information [file srep44154-s1.pdf]
